# Silver nitrate enhances antibacterial effect of colistin against intrinsic colistin resistant *Edwardsiella piscicida*

**DOI:** 10.3389/fvets.2025.1679761

**Published:** 2025-10-29

**Authors:** Yangbin Shi, Luyu Mei, Zubair Ahmed Laghari, Caiyi Tu, Yajing Pan, He Zhang, Yongliang Lou, Jinfang Lu

**Affiliations:** 1 Wenzhou Key Laboratory of Sanitary Microbiology, Key Laboratory of Laboratory Medicine, Ministry of Education, School of Laboratory Medicine and Life Sciences, Wenzhou Medical University, Wenzhou, Zhejiang, China; 2 Department of Veterinary Parasitology, Sindh Agriculture University, Tando Jam, Sindh, Pakistan; 3 Zhejiang Provincial Key Laboratory for Subtropical Water Environment and Marine Biological Resources Protection, National and Local Joint Engineering Research Center of Ecological Treatment Technology for Urban Water Pollution, College of Life and Environmental Sciences, Wenzhou University, Wenzhou, Zhejiang, China

**Keywords:** fish diseases, antimicrobial resistance, silver ions, colistin, synergistic effect

## Abstract

*Edwardsiella piscicida* (*E. piscicida*) has been recognized as an important bacterial pathogen affecting fish, and it is also intrinsically resistant to colistin. *E. piscicida* infects many species of cultured fish and aquatic animals, posing a significant threat to the global aquaculture industry and ecological systems. Therefore, alternative treatment strategies are urgently required to combat *E. piscicida* infections effectively. In this study, the combination of silver nitrate and colistin demonstrated strong bactericidal activity against both *in vitro* and *in vivo*. Silver nitrate significantly reduced the minimum inhibitory concentration (MIC) of *E. piscicida* and enhanced the antibacterial effect of colistin against *E. piscicida*. Moreover, the combination effectively eliminated *E. piscicida* in zebrafish, and significantly increased their survival. Mechanistic analysis revealed that silver nitrate and colistin disrupted bacterial redox homeostasis by targeting the thioredoxin (Trx) system, inducing the over-production of reactive oxygen species (ROS) and malondialdehyde (MDA), suppressing the activities of superoxide dismutase (SOD) and catalase (CAT), and depleting glutathione (GSH), resulting in severe oxidative stress. In addition, silver nitrate strongly enhanced the membrane damage ability of colistin, increased membrane permeability, and decreased membrane potential with obvious morphological damages. The silver nitrate–colistin combination strikingly attenuated the essential pathways involving in drug efflux, cationic antimicrobial peptides resistance (CAMP), and mechanisms related to infection and virulence. These results highlight the potential of the combination of silver nitrate and colistin as an effective treatment strategy against intrinsically colistin-resistant *E. piscicida*.

## Highlights

- Silver nitrate breaks *Edwardsiella piscicida* intrinsic colistin resistance.
- Silver nitrate potentiates colistin’s membrane damage ability and exacerbates oxidative stress.
- Silver nitrate rescues the treatment efficacy of colistin against *Edwardsiella piscicida*.
- Silver nitrate and colistin suppress the expression of genes related to CAMP resistance and virulence pathways.

## 1 Introduction

Aquaculture products have become one of the major sources of high-quality protein for humans. The rapid increase in fish consumption has spurred the rapid development and intensification of the aquaculture industry worldwide (1). While infectious diseases caused by various pathogens pose significant challenges to aquaculture and often result in severe economic losses globally (1, 2). Among these fish pathogens, *E. piscicida* (formerly known as *Edwardsiella tarda*) is a widely distributed and emergent opportunistic bacterial pathogen (3, 4). It has been reported in numerous countries and regions, including China (5), the USA (6) and Egypt (7). *E. piscicida* can infect various freshwater and marine fish species (e.g., *Oreochromis mossambicus*, and *Scophthalmus maximus*), often resulting in high mortality rates (3, 4, 8). Antibiotics are the primarily employed to control aquaculture-related infections, including those caused by *E. piscicida* (9). However, the frequent emergence of multi-drug resistance (MDR) *E. piscicida* strains severely exacerbated both the shortage of effective antimicrobial options and the risk of treatment failure (5–7). Therefore, alternative therapeutic strategies are urgently needed to combat *E. piscicida* infections.

Metal or metalloid [metal(loid)]-based antimicrobials (MBAs) have been widely used as antibacterial agents to combat infections for millennia, such as silver nitrate (AgNO_3_), silver nanoparticles (AgNPs) (10–13). The Food and Drug Administration of America (FDA) approved gold-based drug auranofin has demonstrated potent bactericidal activity against various Gram-positive pathogens (14, 15), and strikingly enhanced the bactericidal activity when combined with antibiotics (16, 17). Bismuth-based drugs also have been repurposed to combine with clinically-relevant antibiotics to eliminate MDR *Pseudomonas aeruginosa* (*P. aeruginosa*) (18). The comparatively low toxicity of these agents to mammalian cells and animals has opened new avenues for their application in biomedicine.

Recent developments have demonstrated the effectiveness of silver (Ag) as a potent antibiotic adjuvant. When combined with certain antibiotics, Ag exhibits enhanced synergistic antibacterial effects against various MDR bacteria (19–21). Specifically, silver nitrate (AgNO_3_) enhanced the activity of gentamicin and tetracycline against MDR Gram-negative bacteria both *in vitro* and *in vivo* without evident toxicity to mammalian cells or mice (19). AgNO_3_ combined with colistin also exhibited broad-spectrum antimicrobial activity against *mcr* positive bacteria (21). Additionally, AgNPs combined with natural bioactive compounds could effectively fight against fish pathogens such as *Aeromonas hydrophila* (*A. hydrophila*), *Vibrio harveyi* (*V. harveyi*) (22–24), and even spring viraemia virus (25). These findings highlight the potential of Ag-based treatments in controlling a wide range of fish pathogens and preventing disease outbreaks in aquaculture.

Colistin (polymyxin E), as a well-known broad-spectrum antibiotic, has been considered as one of the last-resort options for treating infections caused by various MDR Gram-negative bacterial pathogens (26, 27). This cationic antibiotic disrupts bacterial membrane integrity by directly interacting with the lipid A of the outer membrane, leading to leakage of cytoplasmic contents and subsequent cell death (26, 27). However, modifications of lipid A mediated by intrinsic pathways or mobile colistin resistance (*mcr*) variants have further aggravated colistin resistance (26–28), severely compromising colistin’s bactericidal efficacy and contributing to the growing prevalence of colistin-resistant bacteria.

Though *E. piscicida* strains varies with their own antibiotic tolerance spectrums, they are all naturally resistant to colistin. We previously found that AgNO_3_ can effectively re-sensitize *E. piscicida* to colistin. Notably, the combination of AgNO_3_ and colistin exhibits striking synergistic antibacterial activity against MDR *E. piscicida*. However, the underlying mechanism of this synergistic effect remained unclear. The present study investigated the bactericidal effects and elucidated the potential mechanisms of the AgNO_3_-colistin combination. The aim was to support the repurposing of AgNO_3_ as a novel adjuvant to colistin for the effective treatment of bacterial infections caused by colistin-resistant fish pathogens.

## 2 Materials and methods

### 2.1 Bacterial strains and cultivation

*E. piscicida* PPD130/91 was provided by Prof. Haixia Xie (Institute of Hydrobiology, Chinese Academy of Sciences, Wuhan, Hubei, China). LY-2019 and ZX-1 were isolated from diseased fish in our laboratory. All mutants were derived from PPD130/91 (29), and listed in Supplementary Table S1. *In vivo* experiments were conducted with *E. piscicida* PPD130/91. These strains had been identified, and stored in TSB nutrient broth with 20% glycerol at −80 °C in our laboratory.

### 2.2 Checkerboard assay

The synergistic antibacterial effect of colistin and AgNO_3_ against *E. piscicida* isolates were assessed using the checkerboard assay. In brief, colistin and AgNO_3_ were diluted based on the minimum inhibitory concentrations (MIC_colistin_) of each isolate, along the *x*- and *y*-axis of a flat-bottom 96-well microtiter plate to form an 8 × 12 matrix. Overnight bacterial cultures were re-suspended in phosphate-buffered saline (PBS, pH 7.4), and 100 μL of the bacterial suspension was added to each well of a 96-well microtiter plate to achieve a final density of 5.0 × 10^5^ CFU/mL. The microplate was then incubated at 28 °C for 20 h, and the absorbance at OD_540_ was measured using a microplate reader (BioTek, Vermont, United States). The fractional inhibitory concentration index (FICi) was used to assess the synergistic antibacterial effect of colistin and AgNO_3_ as previously described (29, 30). FICi was calculated using the following formula:


$$\text{FICi}=({\mathrm{MIC}}_{a\;\text{combined}\;b}/{\mathrm{MIC}}_{a\;\text{alone}})+({\mathrm{MIC}}_{b\;\text{combined}\;a}/{\mathrm{MIC}}_{b\;\text{alone}})$$

Here, colistin and AgNO_3_ are denoted as “a” and “b,” respectively. FICi values of ≤0.5, 0.5 to 1.0, and >1.0 indicate synergy, an additive effect, and antagonism, respectively. All analyses were performed in three biological replicates.

### 2.3 Time killing assay

A time-dependent killing assay was used to evaluate the synergistic bactericidal activity of colistin/AgNO_3_, and the bacterial growth kinetics following treatment. Briefly, each colistin-resistant *E. piscicida* isolate was inoculated into 25.0 mL of CAMHB medium containing either colistin, AgNO_3_ alone, or a combination of colistin and AgNO_3_, at an initial concentration of 5.0 × 10^5^ CFU/mL. Each group (the untreated control, colistin alone, AgNO_3_ alone, and the combination of colistin plus AgNO_3_) was incubated at 28 °C, and sampled at the indicated time points (1, 3, 5, 7, 9, 12, and 24 h). Bacterial suspensions were plated on TSB agar for colony-forming units (CFU) enumeration after appropriate dilutions. A ≥2 log_10_ reduction in bacterial CFU at 24 h, compared to either single treatment, was considered as indicative of a synergistic bactericidal effect.

### 2.4 Resistance development study

Overnight cultures of *E. piscicida* (OD_540_ ≈ 0.5) were collected and diluted (1:200) in fresh TSB medium. The bacterial cultures were then incubated at 28 °C in TSB medium containing 16.0 μg/mL of colistin (1/4 of MIC), with or without AgNO_3_ (1.0 μg/mL, 1/4 of MIC), for 24 h. The bacterial cultures were serially passaged daily in fresh TSB (1:200) containing the corresponding drug (s), and the MIC for the evolved *E. piscicida* subpopulations was simultaneously assayed over a period of 21 days.

### 2.5 Fluorescence assay

#### 2.5.1 LIVE/DEAD bacterial cell viability assay

A LIVE/DEAD^™^ BacLight^™^ Bacterial Viability Kit was used to assess the viability of *E. piscicida* cells. In brief, *E. piscicida* cells were treated with the indicated concentrations of colistin or AgNO_3_ alone, or in combination, at 28 °C for 2 h. The cells were then stained with PI (50 μg/mL, excitation at 561 nm/emission at 617 nm) and SYTO 9 (100 μg/mL, excitation at 488 nm/emission at 530 nm) following a previous study (29), and the manufacturer’s instructions. Samples were photographed using a phase-contrast fluorescence microscope (Nikon, Tokyo, Japan). Cells exhibiting green fluorescence were considered as viable, indicating an intact membrane, while cell showing red fluorescence were considered as non-viable, indicating a compromised bacterial membrane. The dead-to-live cell ratio was determined by counting the number of cells with red or green fluorescence, and at least 1,000 cells were counted across five representative slides from each treatment, respectively.

#### 2.5.2 Membrane permeability assay

A fluorescent probe, 1-N-phenyl naphthylamine (NPN), was used to assess the outer membrane (OM) permeability of *E. piscicida*. In brief, overnight bacterial cultures were collected and suspended in 5.0 mM HEPES buffer (pH 7.0), and the OD_540_ was standardized to 0.5. The bacterial suspension was then treated with colistin (16.0 μg/mL), AgNO_3_ (1.0 μg/mL), or a combination of both. After incubation at 28 °C for 4 h, the *E. piscicida* cells were mixed with the NPN probe to reach a final concentration of 10.0 μM and incubated at 25 °C for 45 min. Fluorescence intensity was subsequently measured using a BioTek microplate reader (Vermont, United States) (50.0 μg/mL, excitation at 355 nm/emission at 420 nm).

Propidium iodide (PI) staining was used to assess the integrity of the inner membrane (IM) of *E. piscicida*. Overnight cultures were treated using protocols as described above, and then incubated with 10.0 μM PI at 25 °C for 45 min. Finally, fluorescence intensity was measured using a microplate reader (excitation at 561 nm/emission at 615 nm).

#### 2.5.3 Proton motive force assay

The bacterial membrane potential was assessed using the fluorescent probe DiSC_3_(5), according to the manufacturer’s instructions. *E. piscicida* cells were incubated with 5.0 μM DiSC_3_(5) in HEPES buffer (pH 7.4) for 15 min, following a 30 min-treatment with colistin (16.0 μg/mL), AgNO_3_ (1.0 μg/mL), or their combination at 25 °C. Fluorescence intensity was measured using a microplate reader (excitation at 622 nm/emission at 670 nm).

#### 2.5.4 Efflux pump assay

Changes in efflux pump activity were evaluated by monitoring the accumulation of Hoechst 33342 dye. *E. piscicida* cells were first treated with colistin (16.0 μg/mL), AgNO_3_ (1.0 μg/mL), or their combination at 25 °C for 30 min, then stained with 2.5 μM Hoechst 33342. Heat-inactivated bacterial cells were used as the positive control. The fluorescence intensity of accumulated Hoechst 33342 was measured using a microplate reader (excitation at 355 nm/emission at 460 nm).

#### 2.5.5 Reactive oxygen species detection

The DCFH-DA fluorescent probe was used to detect the ROS level in *E. piscicida*. Bacterial cells were treated as described in section 2.5.2, then stained with 5.0 μM of DCFH-DA at 25 °C for 45 min. Finally, fluorescence intensity was measured using a microplate reader (excitation at 488 nm/emission at 525 nm).

### 2.6 Biochemical parameter analysis

The activities of TrxR, CAT, and the contents of MDA and GSH were determined using the corresponding commercial kits, following a previous study (29) and the manufacturer’s instructions. Briefly, bacterial cells were treated as described in section 2.5.2, then each *E. piscicida* culture was harvested, suspended in HEPES buffer, and lysed using an Ultrasonic Cell Crusher on ice (2 s on, 2 s off; 60 W; 20 min). The supernatant was collected by centrifugation at 12,000 rpm at 4 °C for 5 min, and was used for subsequent biochemical parameters analysis. The specific wavelengths for TrxR, CAT, MDA, and GSH measurements were 412, 405, 535 and 412 nm, respectively, using a BioTek microplate reader (Vermont, United States).

### 2.7 Colistin accumulation assay

The colistin accumulation assay was performed according to previous studies (29, 30) and the manufacturer’s instructions. Briefly, the bacteria cells were collected and lysed as described in section 2.6, bacterial supernatant was collected by centrifugation (14,000 rpm, 5 min) for detection of intracellular content of colistin using a Colistin ELISA kit (Abebio, Wuhan, China).

### 2.8 Bacterial biofilm analysis

The inhibitory effect of the combination of colistin and AgNO_3_ on *E. piscicida* biofilm formation was evaluated using crystal violet staining as described in previous study (29). *E. piscicida* cultures (OD_540_ ≈ 0.5) were prepared, then treated with colistin, AgNO_3_ alone, or their combination. After a 24-h incubation at 28 °C, the medium containing planktonic bacteria was removed, and the wells were gently washed with PBS three times. Next, 1.0% of crystal violet solution was added into each air-dried well and incubated for 15 min at 37 °C. After washing three times with PBS and allowing the wells to air dry, 200 μL of decolorizing solution (95% ethanol + 5% acetic acid) was added to each well to dissolve the residual crystal violet at 37 °C for 15 min. The optical absorbance was finally measured at 595 nm using a BioTek microplate reader.

### 2.9 Bacterial motility assay

For the motility assay, 5.0 μL of bacterial cultures treated with colistin, AgNO_3_ alone, or their combination were inoculated at the center of TSB swimming motility agar plates (0.3%, w/v), and allowed to stand undisturbed for 30 min. The plates were then incubated at 28 °C for 16 h, and the diameters of the motility halos were measured to evaluate bacterial swimming motility.

### 2.10 RNA isolation and real-time quantitative PCR analysis

Total RNA was extracted using a Bacteria RNA Extraction Kit (Vazyme, Nanjing, China) following the manufacturer’s instructions. Elimination of contaminated bacterial genomic DNA and reverse transcription (1 μg of total RNA) was carried out with a PrimeScript^™^ RT reagent kit with gDNA Eraser (Takara, Dalian, China). Real-Time Quantitative PCR (RT-qPCR) was then performed using the ArtiCanATM SYBR QPCR Mix (Qingke, Beijing, China) in a CFX Connect^™^ Fluorescent Quantitative PCR Detection System (Bio-Rad, CA, United States). The specific primers used in this study are listed in Supplementary Table S2. The relative expression levels of the target genes were calculated using the 2^−ΔΔCT^ method (31).

### 2.11 Scanning electron microscope

The morphological changes of *E. piscicida* under different treatments were observed using a scanning electron microscope (SEM). Specifically, overnight bacterial cultures were treated with colistin, AgNO_3_ alone, or their combination at 28 °C for 24 h. *E. piscicida* cells were then fixed with 2.5% glutaraldehyde at 4 °C for 24 h. Following fixation, the bacterial cells were gradually dehydrated using a graded ethanol series (30, 50, 70, 90, and 100%). Finally, the samples were dried using a critical point dryer, coated with a layer of gold–palladium, and observed under a SEM (JEOL, Tokyo, Japan).

### 2.12 Zebrafish infection model

The zebrafish infection model was established as described in previous studies (29). Briefly, well grown, clinically healthy, uniform-sized adult fish (~6 months old, gender-neutral, 0.4 ± 0.05 g) were randomly divided into five groups (*n* = 20). Group 1 served as the negative control was just treated with PBS only. Group 2–5 were infected with an exponential-phase *E. piscicida* suspension (1.0 × 10^4^ CFU). At 2 h post-infection, group 2 received a single intraperitoneal dose of PBS; groups 3 and 4 were treated with colistin (8.0 mg/kg) or AgNO_3_ (1.5 mg/kg) alone, respectively; group 5 received a combination of colistin (8.0 mg/kg) and AgNO_3_ (1.5 mg/kg). At 48 h post-infection, five zebrafish from each group were humanely euthanized with the rapid cooling method in an ice–water bath (2–4 °C) (32). Then the liver, spleen, kidney, intestine, and gill tissues were collected and homogenized for colony counting to determine the bacterial load. Survival rates were monitored and recorded daily for 14 days. Zebrafish infection and treatment assays were conducted by authors YP, CT, YS, and LM blinded to group division conducted quantification of bacterial loads in zebrafish organs and survival rates. Microscopic examination of infected fish with typical clinical symptoms (e.g., swollen and hyperaemic muscles) was recruited as a key criterion to confirm the success of bacterial infection.

The administered doses of colistin (8 mg/kg) and AgNO₃ (1.5 mg/kg) were based on several studies (19, 21) (Supplementary Table S3), and our preliminary results. Morones-Ramirez et al. (19) and Zhang et al. (21) observed no apparent toxicity of AgNO_3_ at administered doses of 6 mg/kg and 1.5 mg/kg in mice, respectively. Moreover, a number of studies reported that 1.5–10.0 mg/kg of colistin is safe and feasible for *in vivo* assays (Supplementary Table S3). Moreover, our preliminary found that 1.5 mg/kg of AgNO_3_ did not caused any apparent toxicity to zebrafish, and can mostly improve the survival rates of infected fish with 8.0 mg/kg of colistin. Hence, the doses of colistin (8 mg/kg) and AgNO₃ (1.5 mg/kg) was used in the present study.

### 2.13 Statistical analyses

Data are presented as mean ± SD. GraphPad Prism 9.5.0 was used for statistical analysis. Unless stated otherwise, normally distributed data were analyzed using an unpaired *t*-test for comparisons between two groups, and one-way analysis of variance (ANOVA) for multiple group comparisons. The log-rank test was performed to assess the statistical significance of survival data from *in vivo* studies. In case where the data were not normally distributed, the Mann–Whitney *U* test was used to calculate *p*-values (^*^p < 0.05, ^**^p < 0.01, and ^***^p < 0.001).

## 3 Results

### 3.1 Ag mediates reversal of colistin resistance in *Edwardsiella piscicida*

**Figure 1:**
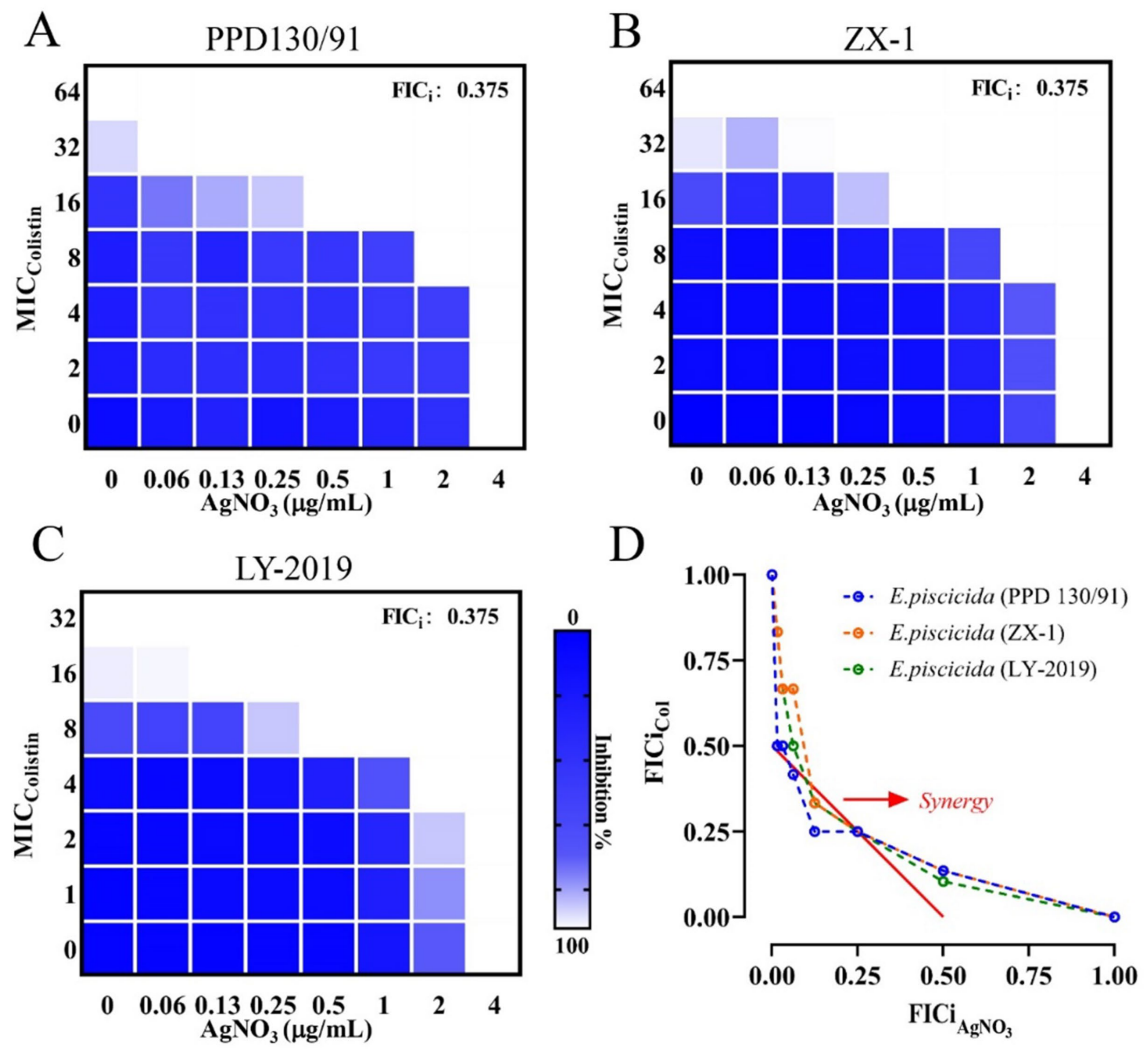
Synergistic effect of AgNO_3_ and colistin against three *E. piscicida* isolates. Representative heat maps of checkerboard assays for AgNO_3_ combined with colistin against *E. piscicida* isolates. **(A)** PPD130/91, **(B)** ZX-1, and **(C)** LY-2019. The darker blue areas represent greater bacterial cell density. **(D)** Isobolograms of the combination of AgNO_3_ and colistin against three *E. piscicida* isolates. The red full line indicates ideal isobole, and data points below it (<0.5) represent synergy. The checkerboard and FICi data are from at least three biological replicates (*n* = 3).

The combination of AgNO_3_ and colistin exhibited striking synergistic activity against *E. piscicida*. Specifically, the FICi values for strains PPD130/91, ZX-1, and LY-2019 were 0.375 (<0.5) (Figure 1 and Table 1). The MIC_colistin_ was reduced by four-fold from 64.0 to 16.0 μg/mL for both PPD130/91 and ZX-1, and from 32.0 to 8.0 μg/mL for LY-2019 (Table 1). These results suggest that AgNO_3_ treatment can effectively overcome the intrinsic resistance of *E. piscicida*.

**Table 1:** FICi values for colistin/silver nitrate combinations against three selected *E. piscicida* isolates and *trx* mutants.

Strains	AgNO_3_ (μg mL^−1^) MIC_com_/MIC_alone_	Colistin (μg mL^−1^) MIC_com_/MIC_alone_	FIC index	Interpretation
*E. piscicida* PPD130/91	0.5/4	16/64	0.375	Synergy
*E. piscicida* ZX-1	0.5/4	16/64	0.375	Synergy
*E. piscicida* LY-2019	0.5/4	8/32	0.375	Synergy
*E. piscicida* PPD130/91 + NAC	64/1024	32/64	0.563	Additive
*E. piscicida* ZX-1 + NAC	64/1024	64/128	0.563	Additive
*E. piscicida* LY-2019 + NAC	64/1024	64/128	0.563	Additive
Δ*trxA*	0.5/4	16/64	0.375	Synergy
Δ*trxB*	1/4	32/64	0.750	Additive
Δ*trxC*	0.5/4	32/64	0.625	Additive
Δ*trxA*, Δ*trxC*	1/4	32/64	0.750	Additive
Δ*trxA*, Δ*trxB*, Δ*trxC*	2/4	32/64	1.000	Additive

**Figure 2:**
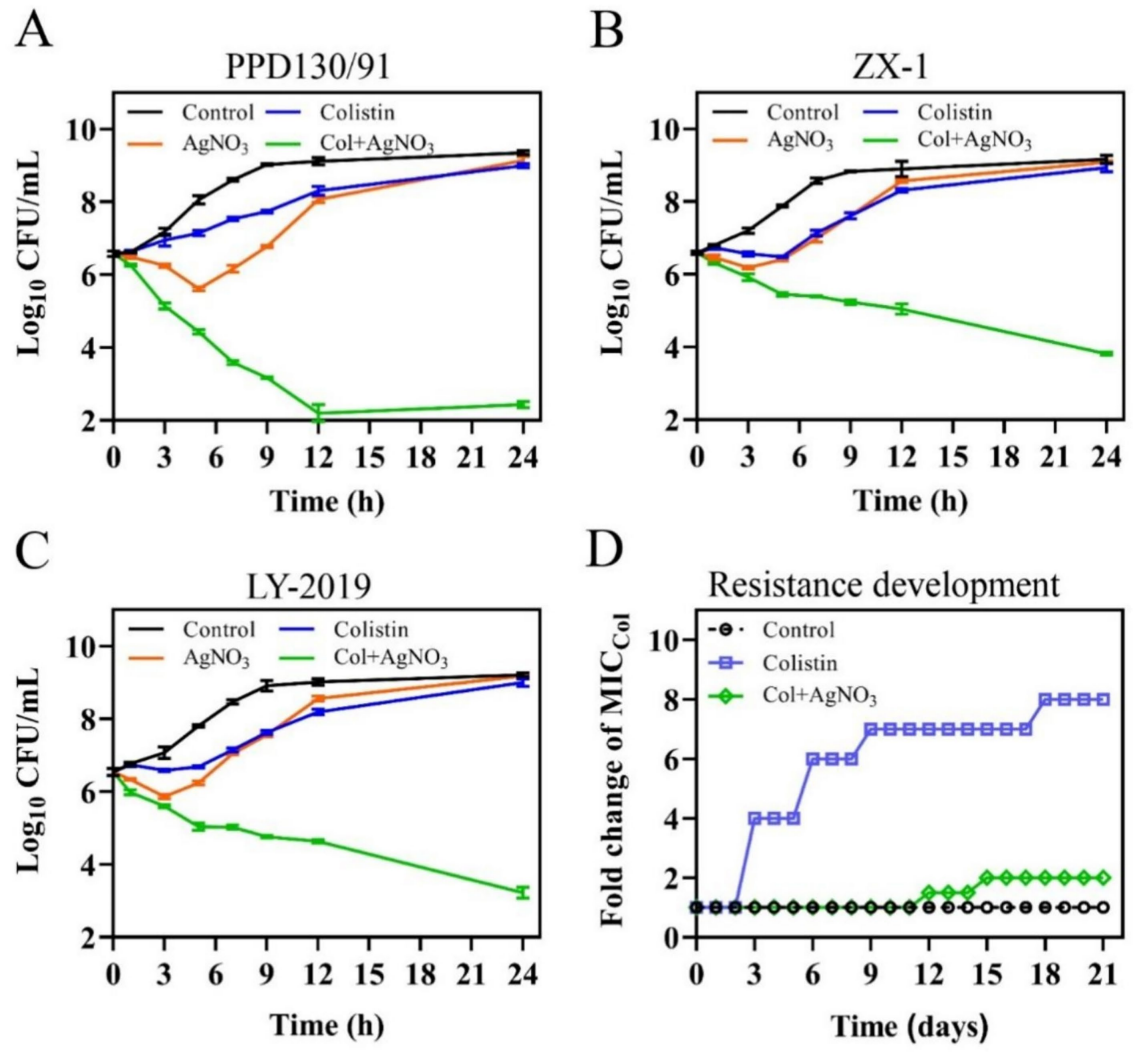
AgNO_3_ restores the sensitivity of naturally colistin resistant *E. piscicida* to colistin and suppresses the evolution of colistin resistance. **(A–C)** Time-dependent kill curves of three colistin-resistant *E. piscicida* isolates by the combination of AgNO_3_ (1.0 μg/mL), colistin (col, 16.0 μg/mL). **(A)** PPD130/91, **(B)** ZX-1, and **(C)** LY-2019. **(D)** Resistance acquisition curve of *E. piscicida* PPD130/91 in the presence of colistin alone or in combination with AgNO_3_ during a 21-day serial passage. The values are expressed as mean ± SD (*n* = 3).

Time-kill assays revealed that the combination of AgNO_3_ with 8.0–16.0 μg/mL colistin exhibited enhanced bactericidal activity, significantly reducing the colony counts of *E. piscicida* strains by 5.87–6.92, 3.27–3.86, and 3.56–4.38 log10 CFU/mL at 12 h, and by 6.56–6.91, 5.12–5.36, and 5.78–5.98 log10 CFU/mL at 24 h for PPD130/91, ZX-1, and LY-2019, respectively (Figures 2A–C), compared to the control and monotherapy groups. Moreover, no bacterial re-growth was observed in the combination treatment group at 24 h post-treatment (Figures 2A–C). In contrast, treatment with either colistin or AgNO_3_ alone only slightly inhibited the growth of the three isolates during the early incubation phase (0–6 h), followed by noticeable re-growth (Figures 2A–C). Collectively, these findings demonstrated that AgNO_3_ not only reversed the intrinsic colistin resistance of *E. piscicida*, but also acted synergistically with colistin.

### 3.2 Ag suppresses development of colistin resistance and biofilm formation

The resistance development assays detected a stable increase in the MIC_colistin_ for *E. piscicida* when treated with colistin alone. The MIC_colistin_ value reached 512.0 μg/mL by day 21, representing an 8-fold increase compared to the control group (Figure 2D). Exogenous supplementation with 1.0 μg/mL AgNO_3_ substantially slowed the development of colistin resistance, with the MIC_colistin_ increasing to only 128.0 μg/mL by day 21, representing a 2-fold increase (Figure 2D). These results imply that AgNO_3_ can effectively suppress the development of colistin resistance in *E. piscicida*.

**Figure 3:**
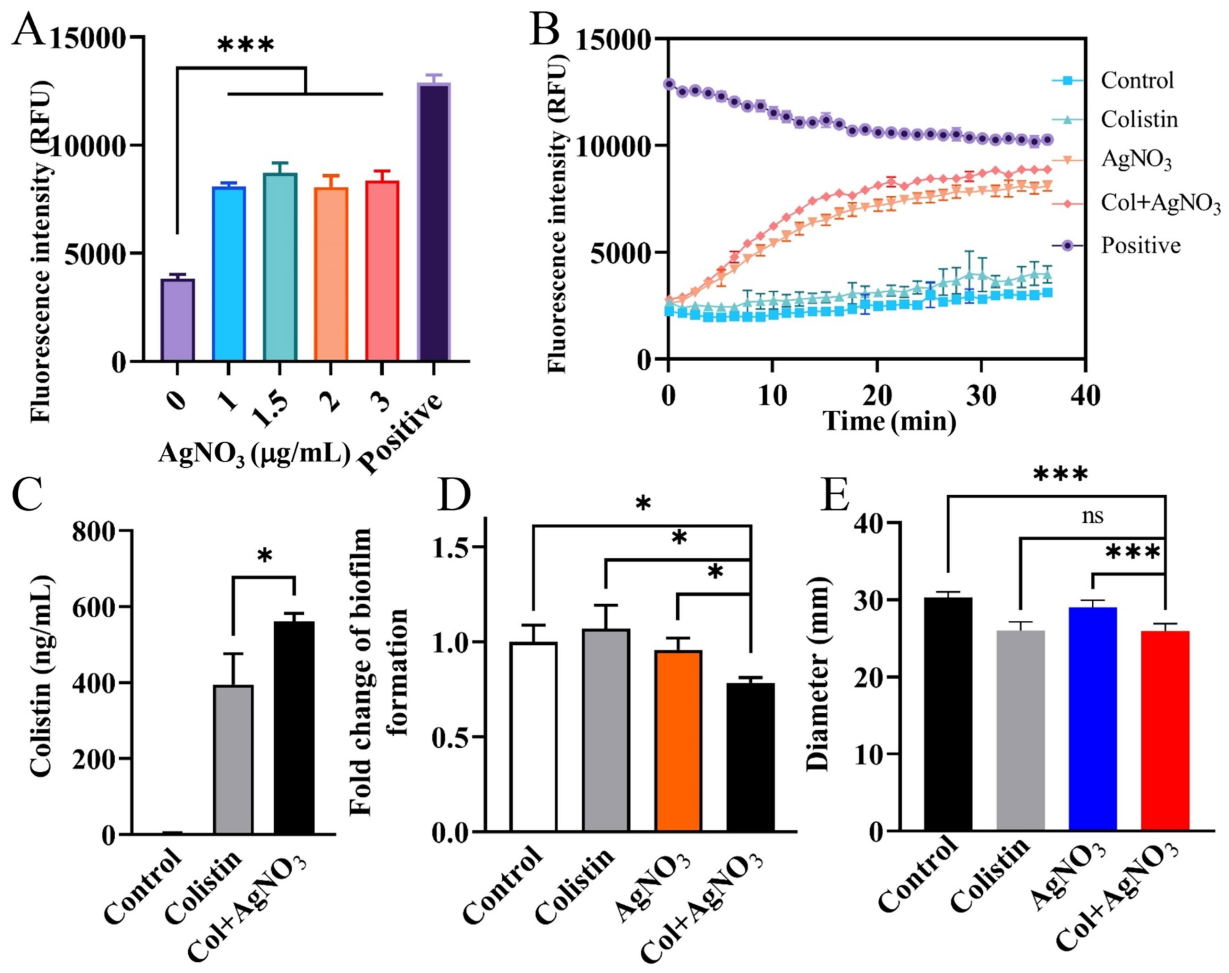
AgNO_3_ enhances efflux pump inhibition, facilitates intracellular colistin accumulation, and reduces biofilm formation and motility of *E. piscicida*. **(A,B)** AgNO_3_ inhibits the efflux activity of *E. piscicida*. The efflux activity was assessed by detection of the accumulation of Hoechst 33342. The heat-inactivated cells were used as positive control. **(C)** AgNO_3_ increases accumulation of colistin in *E. piscicida* PPD130/91. **(D)** AgNO_3_ combined with colistin inhibits biofilm formation of *E. piscicida* PPD130/91. **(E)** AgNO_3_ combined with colistin inhibits the motility of *E. piscicida* PPD130/91. The values are expressed as mean ± SD (*n* = 4), and statistical differences were tested by one-way ANOVA analysis. (^*^p < 0.05 and ^***^p < 0.001).

Treatment with either AgNO_3_ or colistin alone had no inhibitory effect on biofilm formation. In contrast, the combination of AgNO_3_ and colistin significantly inhibited biofilm formation by 21.53% (*p* < 0.05) compared to the control group (Figure 3D). Since bacterial motility is closely related to biofilm formation, the changes in average motility zone diameters were examined. Values of 30.33, 26.00, 29.00, and 25.83 mm were recorded for the control, AgNO_3_ alone, colistin alone, and AgNO_3_ + colistin treatment groups, respectively (Figure 3E), with statistically significant differences observed between the AgNO_3_ alone group and the combination treatment group. The combination treatment also significantly altered the mRNA level of the flagellar-related gene *flhD* (Supplementary Figure S1A). These results indicate that AgNO_3_ combined with colistin inhibits the biofilm formation of *E. piscicida*, possibly associated with the changes in bacterial motility.

### 3.3 Ag potentiates colistin-mediated membrane damage

**Figure 4:**
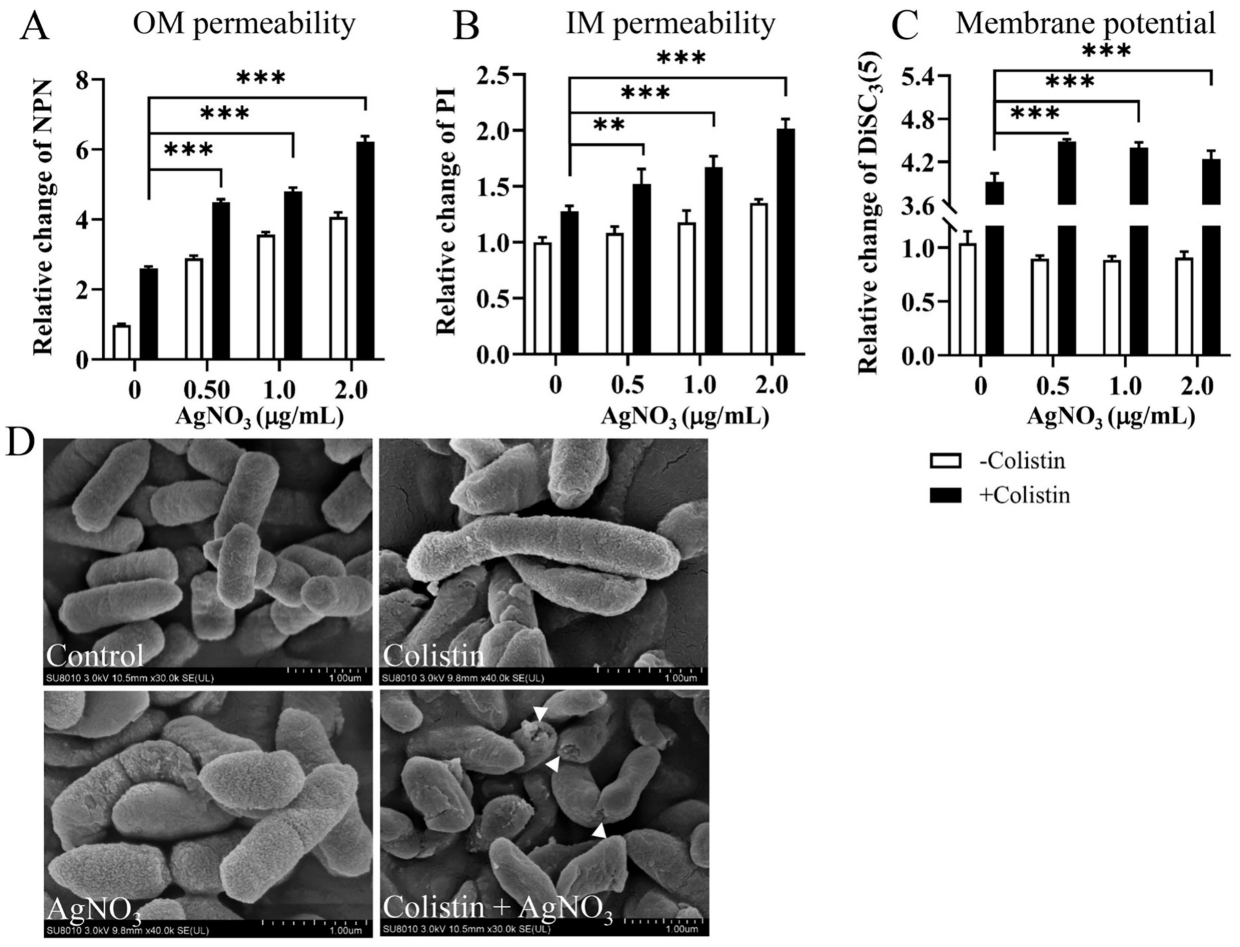
AgNO_3_ potentiates colistin’s membrane damage activity and dissipates the proton motive force. **(A)** AgNO_3_ potentiates the outer membrane (OM) permeability. **(B)** AgNO_3_ potentiates the inner membrane (IM) permeability. **(C)** AgNO_3_ dissipates the proton motive force (PMF). The fluorescence intensities of NPN, PI and DiSC_3_(5) were, respectively, used to evaluate the OM permeability, IM permeability and PMF after exposure to increasing concentrations of AgNO_3_ with constant colistin. **(D)** Morphological observation of *E. piscicida* PPD130/91 after exposure to colistin, AgNO_3_, or colistin + AgNO_3_. Scar bar, 1.0 μm. The values are expressed as mean ± SD (*n* = 4), and statistical differences were tested by two-way ANOVA analysis. (^**^p < 0.01 and ^***^p < 0.001).

Outer membrane (OM) permeability was determined using the NPN hydrophobic fluorescent probe. The fluorescence intensity increased in a dose-dependent manner when AgNO_3_ (0–2.0 μg/mL) combined with colistin (16.0 μg/mL) (Figure 4A). OM permeability gradually potentiated by 164.46, 356.33% (*p* < 0.001), 388.26% (*p* < 0.001), and 532.23% (*p* < 0.001) as the concentration of AgNO_3_ increased from 0, 0.5, 1.0 to 2.0 μg/mL, respectively, compared to the control group. Inner membrane permeability was evaluated using a PI probe. The fluorescence intensity was also markedly enhanced by 27.54, 52.02% (*p* < 0.001), 67.27% (*p* < 0.001), and 101.50% (*p* < 0.001), respectively, at the same increasing concentrations of AgNO_3_ when combined with 16.0 μg/mL of colistin (Figure 4B). Notably, limited increase in fluorescence was observed when *E. piscicida* PPD130/91 cells were exposed to colistin alone (Figures 4A,B). Similar results were observed in *E. piscicida* ZX-1 (Supplementary Figures S2A,B) and LY-2019 (Supplementary Figures S2D,E).

Morphological changes in *E. piscicida* were examined by SEM. As shown in Figure 4D, extensive membrane damage, including cell shrinkage and collapse with holes, were observed in the AgNO_3_ + colistin group, whereas no obvious morphological changes were evident in the control or monotherapy groups (Figure 4D). These data collectively suggest that AgNO_3_ significantly potentiates the membrane-damaging ability of colistin, and they act synergistically against *E. piscicida*.

**Figure 5:**
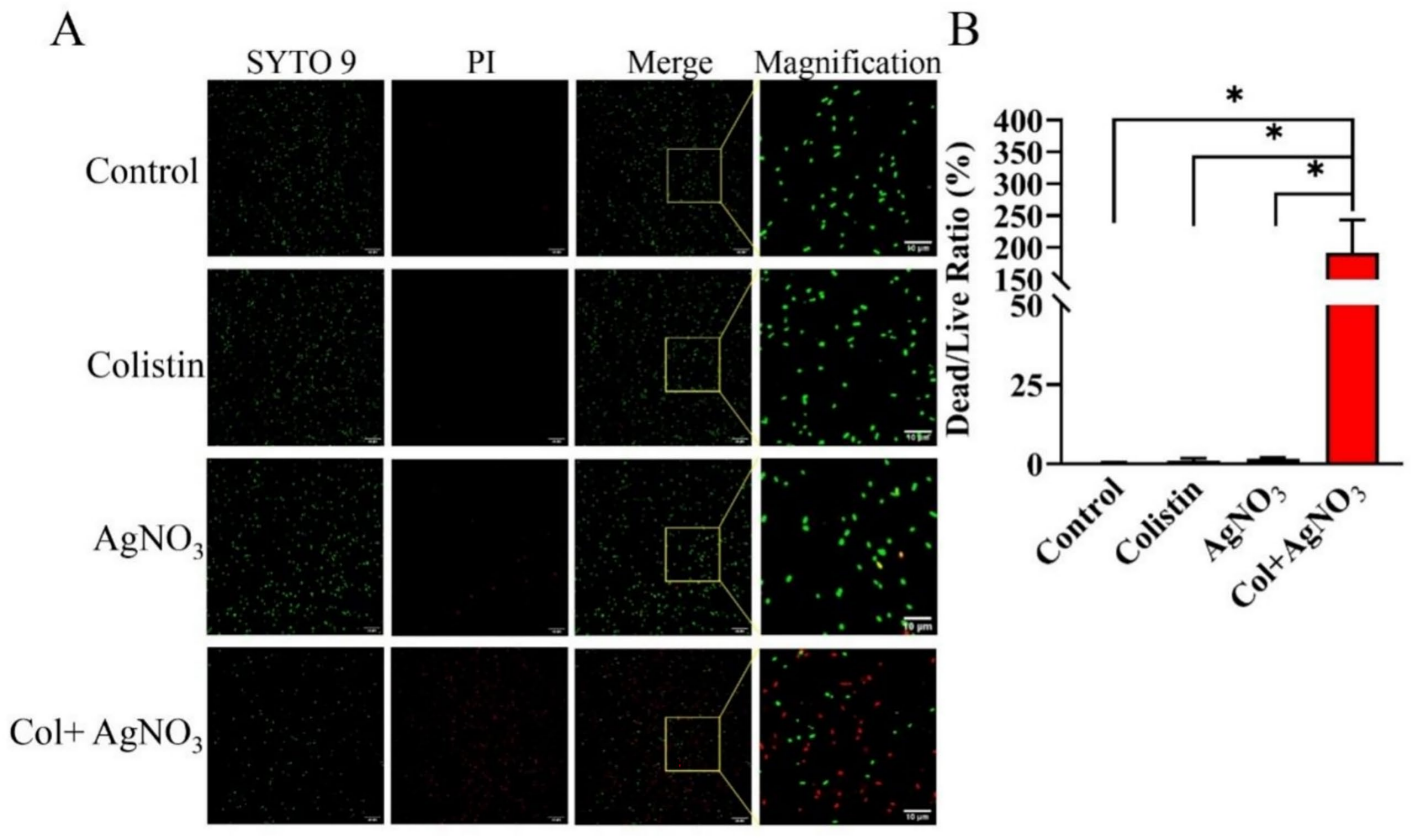
LIVE/DEAD staining to depict the synergistic effect of AgNO_3_ and colistin against *E. piscicida*. **(A)** Representative fluorescence images of strain PPD130/91 after exposure to the colistin and/or AgNO_3_ using two-color dyes Syto9 and PI from the LIVE/DEAD bacterial cell viability kit. **(B)** Dead/live ratio of strain PPD130/91 after exposure to the colistin, AgNO_3_, or colistin + AgNO_3_ for 1 h. The values are expressed as mean ± SD (*n* = 4), and statistical differences were tested by one-way ANOVA analysis. (^*^p < 0.05).

The membrane-damaging effect of AgNO_3_ and colistin was further evaluated using the LIVE/DEAD bacterial cell viability assay. In the control and colistin treatment groups, *E. piscicida* PPD130/91 cells exhibited intense green fluorescence (Figure 5A), with a similar dead/live ratio (Figure 5B), indicating that most bacteria survive with intact membrane. Notably, a small number of cells fluoresced red in the AgNO_3_ treatment group (Figure 5A), indicating that AgNO_3_ has limited antibacterial activity against *E. piscicida*. However, the combination of AgNO_3_ and colistin caused a sharp increase in red fluorescence accompanied by a marked decrease in green fluorescence (Figure 5A).

### 3.4 Ag inhibits efflux activity and dissipates proton motive force

**Figure 6:**
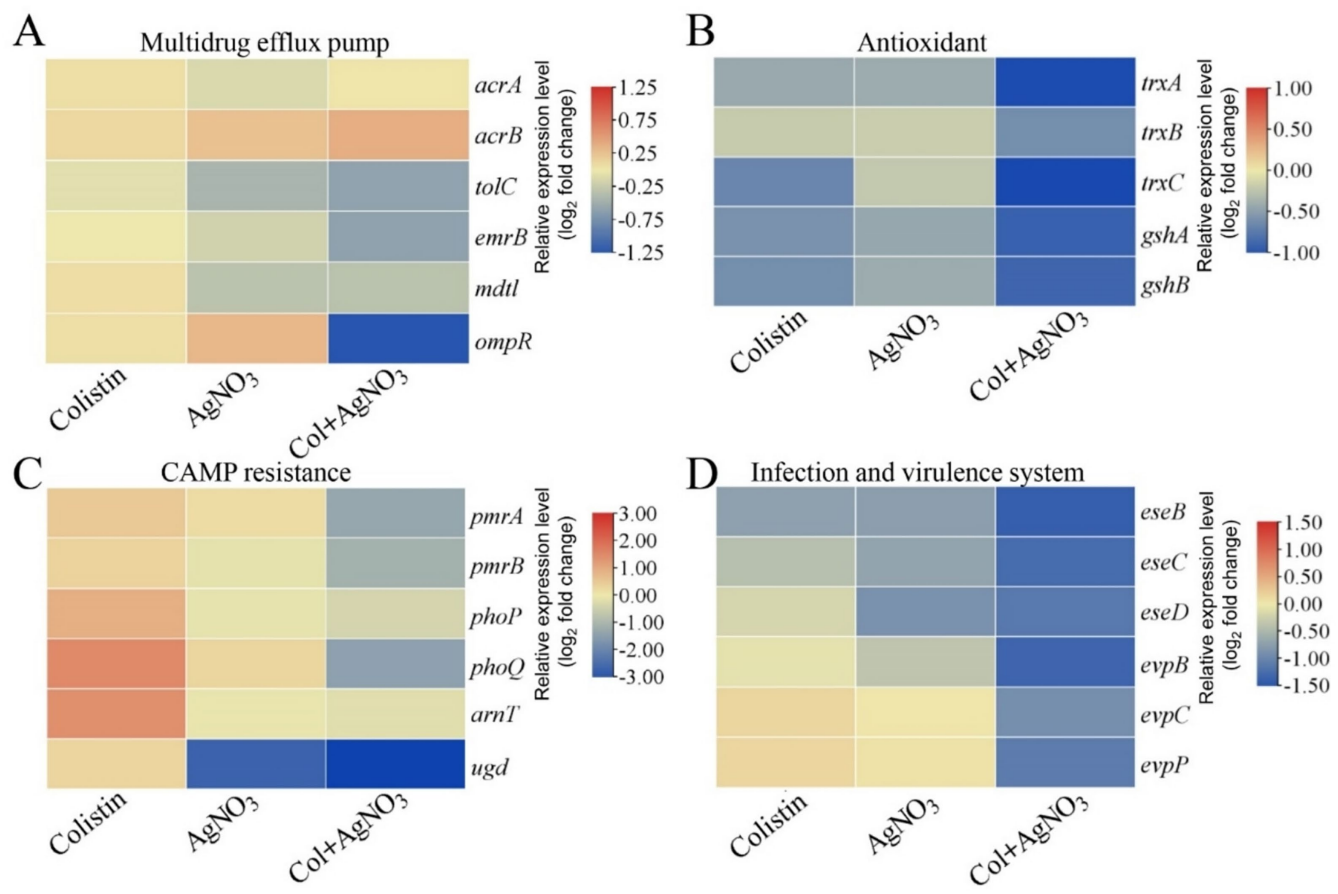
Transcriptional changes of *E. piscicida* treated by AgNO_3_ and colistin. Selected pathways involved **(A)** multidrug efflux pump, **(B)** antioxidant, **(C)** CAMP resistance, and **(D)** bacterial infection and virulence system.

AgNO_3_ alone significantly inhibited efflux activity (Figure 3A). The combination of AgNO_3_ and colistin exhibited stronger inhibition, which was 124.19% (*p* < 0.001) and 9.72% (*p* < 0.01) higher than that of AgNO_3_ alone, respectively (Figure 3B). Correspondingly, the intracellular colistin content increased significantly by 42.04% in PPD130/91 cells receiving the combination treatment (Figure 3C). Similar changes in efflux activity and colistin accumulation were observed in ZX-1 and LY-2019 (Supplementary Figure S3). Consistent with these findings, AgNO_3_ combined with colistin remarkably downregulated three efflux related genes *ompR*, *tolC*, and *emrB* expression by 58.60% (*p* < 0.001), 34.65% (*p* < 0.05), and 34.90% (*p* < 0.05), respectively (Figure 6A). Interestingly, the intracellular content of silver ion (Ag^+^) was reduced by 28.63% (*p* < 0.01) in the combination treatment group compared to the AgNO_3_ alone group (Supplementary Figure S5A).

Additionally, AgNO_3_ combined with various concentrations of colistin markedly increased the fluorescence intensity of the DiSC_3_(5) dye in *E. piscicida* PPD130/91 (Figure 4C). Whereas, neither AgNO_3_ nor colistin alone significantly affected fluorescence intensity. Similar results were observed in ZX-1 and LY-2019 (Supplementary Figures S2C,F). These results suggest that AgNO_3_ combined with colistin effectively inhibits the efflux activity and reduces the membrane potential.

### 3.5 AgNO_3_ combined with colistin promotes oxidative damage

**Figure 7:**
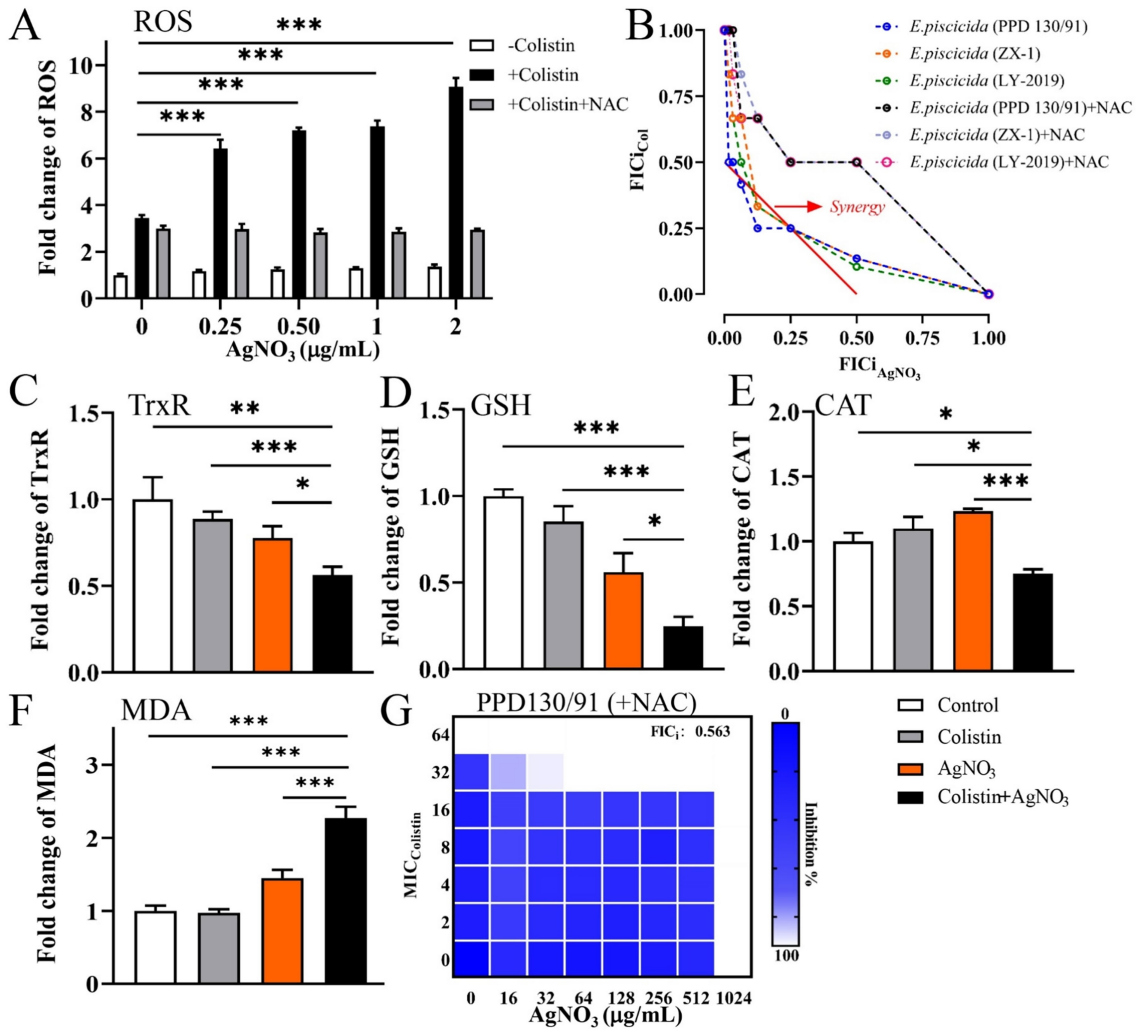
Oxidative stress contributes to the killing efficacy of AgNO_3_ and colistin against *E. piscicida*. **(A)** Relative contents of ROS in *E. piscicida*, respectively, treated by colistin, AgNO_3_, colistin + AgNO_3_, or colistin + AgNO_3_ + NAC. **(B)** Isobolograms of *E. piscicida* isolates treated by colistin + AgNO_3_ + NAC. **(C)** TrxR activity in treated *E. piscicida*. **(D)** Content of GSH in treated *E. piscicida*. **(E)** CAT activity in treated *E. piscicida*. **(F)** Content of MDA in treated *E. piscicida*. **(G)** Isobolograms of *E. piscicida* PPD130/91 treated by colistin + AgNO_3_ + NAC. The values are expressed as mean ± SD (*n* = 4), and statistical differences were tested by one- or two-way ANOVA analysis. (^*^p < 0.05, ^**^p < 0.01, and ^***^p < 0.001).

Treatment with AgNO_3_ alone resulted in only a 1.18–1.37-fold increase in ROS levels compared to the untreated control group. In contrast, significantly higher ROS levels were observed following combination treatment, with the increases of 541.70% (*p* < 0.001), 620.00% (*p* < 0.001), 638.40% (*p* < 0.001), and 807.90% (*p* < 0.001) corresponding to AgNO_3_ concentrations of 0.25, 0.5, 1.0, and 2.0 μg/mL, respectively (Figure 7A). ROS also significantly increased in *E. piscicida* ZX-1 (Supplementary Figure S6A) and LY-2019 (Supplementary Figure S6B) underwent similar treatments.

Other oxidative stress parameters were also examined. Treatment with colistin alone did not significantly affect TrxR activity, while AgNO_3_ alone or its combination with colistin caused marked reductions in TrxR activity by 22.30 and 43.86% (*p* < 0.01), respectively, compared to the control group (Figure 7C). The GSH contents were reduced by 14.71, 43.94, and 75.25% (*p* < 0.001) in the colistin alone, AgNO_3_ alone, and combination treatment groups, respectively, compared to the control group (Figure 7D). CAT activity remained unchanged with colistin alone but increased by 23.39% following AgNO_3_ treatment. In contrast, the combination of AgNO_3_ and colistin significantly reduced CAT activity by 24.67% (*p* < 0.05) (Figure 7E). The MDA contents increased by 44.77% with AgNO_3_ alone and by 126.99% (*p* < 0.001) with the combination treatment (Figure 7F). These results demonstrate that AgNO_3_ and colistin synergistically enhanced oxidative stress in *E. piscicida*.

To further investigate the role of oxidative stress in this synergism, 5.0 mM of N-Acetyl-L-cysteine (NAC), a ROS scavenger, was added to the ROS detection assays. The ROS levels were reduced by 53.72, 60.67, 61.19, and 67.45% in the combination treatments containing 0.25, 0.5, 1.0, and 2.0 μg/mL of AgNO_3_, respectively (Figure 7A). NAC supplementation also significantly alleviated the bactericidal effect of AgNO_3_ and colistin in the checkerboard assays, and increased all FICi values for the three *E. piscicida* strains to 0.563 (Figures 7B,G; Supplementary Figure S4). These findings indicate the critical role of oxidative stress in the synergism activity of AgNO_3_ and colistin.

**Figure 8:**
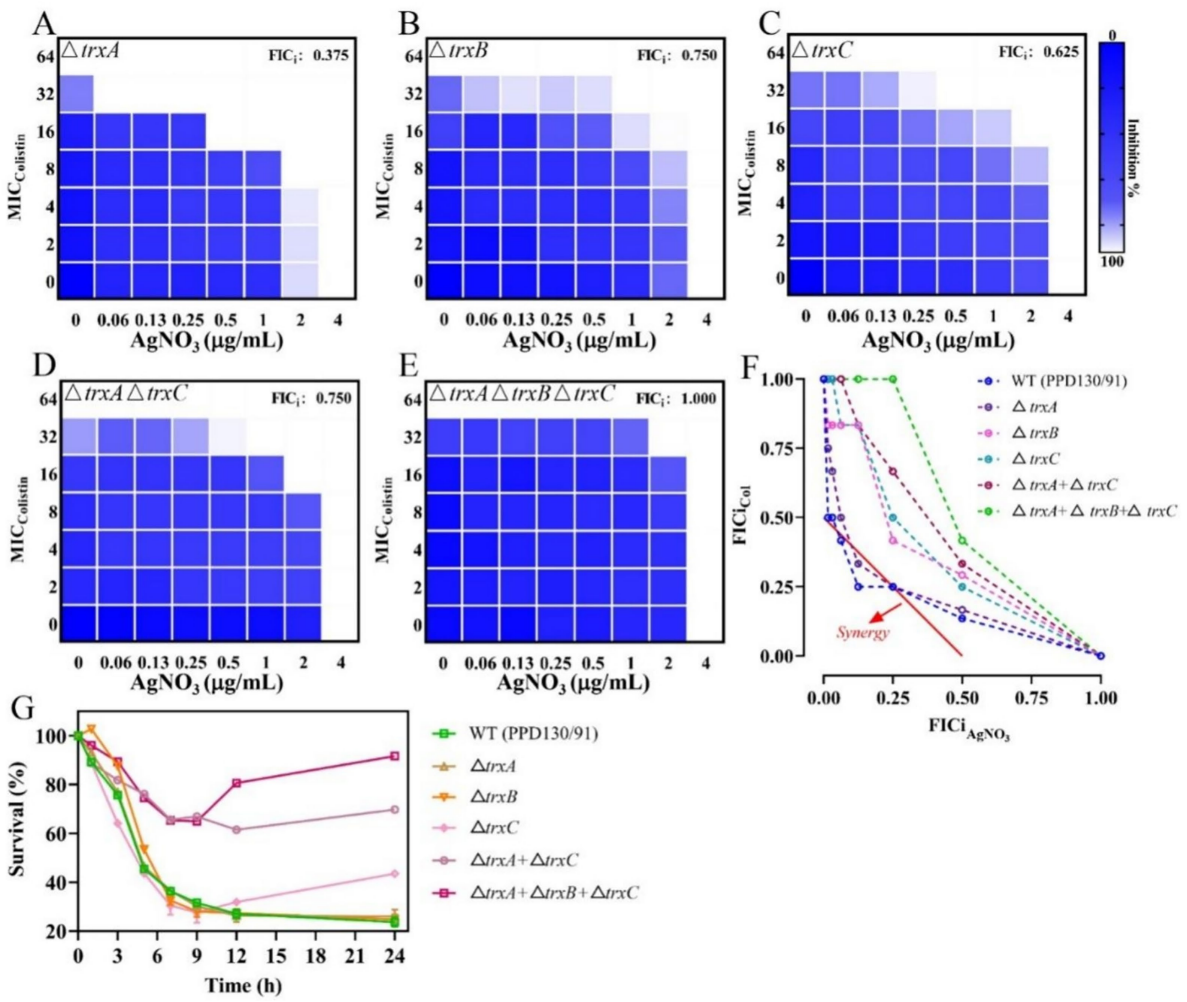
Inactivation of *trx* genes mitigates the synergistic effect of AgNO_3_ and colistin on *E. piscicida*. **(A–E)** Checkerboard assays between AgNO_3_ and colistin in *E. piscicida trx* mutants. **(F)** Isobolograms of *E. piscicida trx* mutants treated by colistin + AgNO_3_. **(G)** Survival ratio of *E. piscicida trx* mutants after exposure to colistin + AgNO_3_. The values are expressed as mean ± SD (*n* = 3).

The expression levels of the antioxidant-related genes were substantially downregulated following treatment with AgNO_3_ and colistin. Specifically, *trxA*, *trxB*, *trxC*, *gshA*, and *gshB* expression decreased by 12.00–37.75% under individual treatments. They were notably suppressed by 52.67% (*p* < 0.001), 35.13% (*p* < 0.001), 53.67% (*p* < 0.001), 48.06% (*p* < 0.01) and 46.49% (*p* < 0.001), respectively, with the combination treatment compared to the control group (Figure 6B). Inactivation of *trxB*, *trxC*, both *trxA* and *trxC*, or the simultaneous deletion of *trxA-B-C* attenuated the synergistic effect of AgNO_3_ and colistin, resulting in the increase of FICis values to 0.75, 0.625, 0.75, and 1.0, respectively (except Δ*trxA*, which had an FICi of 0.375) (Figure 8 and Table 1). Results from the time-dependent killing assay also showed that deletion of these antioxidant related genes significantly improved bacterial survival (Figure 8G), especially in the triple mutant (*ΔtrxA-B-C*). These findings indicate that the bacterial antioxidant defense system is a key target of the AgNO_3_ and colistin combination treatment.

### 3.6 The combination of Ag and colistin impairs LPS modification and attenuates bacterial virulence

qRT-PCR analysis revealed that treatment with colistin alone significantly induced the expression of genes related to LPS modification, with *pmrA*, *pmrB*, *phoP*, *phoQ*, *arnT*, and *ugd* upregulated by 42.54% (*p* < 0.05), 29.21% (*p* < 0.05), 92.25% (*p* < 0.001), 188.56% (*p* < 0.001), 171.07% (*p* < 0.01), and 22.88%, respectively (Figure 6C). In contrast, treatment with silver (AgNO_3_) alone slightly inhibited their expression. However, the combined treatment with AgNO_3_ and colistin significantly suppressed the transcription of these genes by 72.77% (*p* < 0.001), 65.52% (*p* < 0.001), 60.99% (*p* < 0.01), 87.76% (*p* < 0.001), 68.23% (*p* < 0.01), and 90.43% (*p* < 0.001), respectively, compared to the colistin-only group (Figure 6B). Similarly, the transcription of genes related to the bacterial virulence pathway (e.g., type III and VI secretion systems) were only slightly affected by colistin or AgNO_3_ alone, but the expression levels of *eseB*, *eseC*, *eseD*, *eveB*, *evpC*, and *evpP* were remarkably reduced by 63.30% (*p* < 0.001), 59.11% (*p* < 0.01), 55.80% (*p* < 0.01), 61.00% (*p* < 0.001), 48.13% (*p* < 0.05), and 53.33% (*p* < 0.01), respectively, following the combined treatment (Figure 6D). These findings suggest that the combination of AgNO_3_ and colistin not only targets colistin resistance pathways but also significantly impairs bacterial virulence systems in *E. piscicida*.

### 3.7 Ag enhances bactericidal effect of colistin *in vivo*

**Figure 9:**
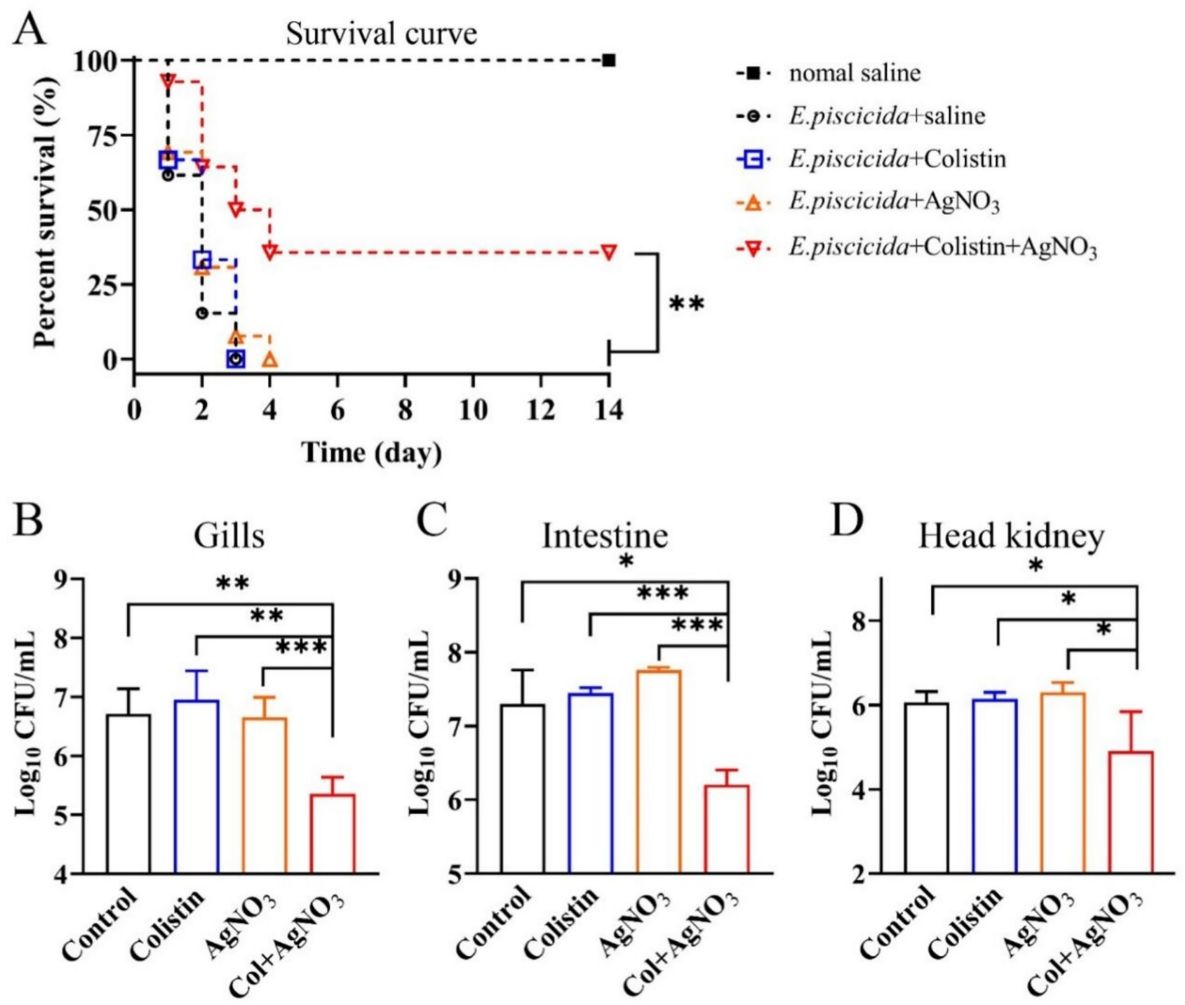
AgNO_3_ potentiates the bactericidal efficacy of colistin *in vivo*. **(A)** Survival curve of *E. piscicida* infection zebrafish (*n* ≥ 9) treated by colistin (8.0 mg/kg), AgNO_3_ (1.5 mg/kg), or their combination (8.0 + 1.5 mg/kg). **(B–D)** Bacterial burdens in the gill, intestine, and head kidney of a zebrafish infection model 48 h post infection with different treatments (*n* = 5). The statistical differences of zebrafish survival rates and bacterial loads were calculated by log-rank (Mantel–Cox) test, and Mann–Whitney *U* test, respectively. (^*^p < 0.05, ^**^p < 0.01, and ^***^p < 0.001).

A zebrafish infection model of *E. piscicida* was established to verify the *in vivo* bactericidal effect of the combination of AgNO_3_ and colistin. For the control and monotherapy groups, extensive mortality was observed with no survivors by day 4 (Figure 9A). In contrast, the combination treatment significantly reduced mortality, improving the survival rate to 35.71% (*p* < 0.01), which was maintained to the endpoint at day 14 (Figure 9A). However, neither normal saline nor monotherapy with AgNO_3_ or colistin alone was sufficient to eradicate *E. piscicida* in zebrafish, and no statistically significant difference was observed among these groups (Figures 9B–D). The combination of AgNO_3_ and colistin significantly reduced the bacterial loads in the gill, intestine, and head kidney by 1.36 (*p* < 0.01), 1.10 (*p* < 0.001), and 1.15 (*p* < 0.05) log10 CFU/mL on day 3, respectively, compared to the colistin monotherapy group (Figures 9B–D). No significant difference was observed in bacterial load in the liver and spleen (Supplementary Figure S7). These data demonstrate that AgNO_3_ enhances the bactericidal activity of colistin *in vivo*, highlighting the potential of combining AgNO_3_ with colistin for the prevention and control of infections caused by colistin-resistant pathogens in aquaculture.

## 4 Discussion

Antibiotics are extensively used to manage pathogens and prophylactically improve animal performance (9, 33). Thereby, the global antimicrobial usage is projected to increase from 10,259 tons in 2017 to 236,757 tons in 2030, and 5.7% of which will be utilized in aquaculture (34). The continuous overuse of antibiotics plays crucial roles in the present antibiotic resistance crisis. Many other countries have recognized the negatively impacts on aquaculture production, the aquatic environments, and human health (35, 36), and a series of strict regulation policies on antibiotics misuse in aquaculture and livestock farming have been released, including the regulation of several antibiotics (e.g., norfloxacin) used for animal infectious diseases, and prohibition of the production of eight growth-promoting antibiotics. While, it is urged to develop more feasible alternative measures to substitute for the old antibacterial strategies.

Several recent studies have reported the potential value of metalloantibiotics in combating bacterial antibiotics resistance. Metal-based strategies have thus emerged as promising strategies to combat MDR pathogens (11, 12, 37). Specifically, the silver complexes, gold metallic drug auranofin, and bismuth-based drugs exhibit strong antibacterial activity, and which remarkably enhanced bactericidal effects when combined with antibiotics (18–20). Notably, due to the outstanding antibacterial property, silver has been approved as a topical antimicrobial by FDA (38). Utilization of silver-based compounds have been remarkably elevated during the last decade (12, 39), including application in aquaculture.

This study demonstrated that the combination of AgNO_3_ and colistin effectively restored the susceptibility of MDR *E. piscicida* to colistin and promoted bacterial eradication *in vitro* (Table 1 and Figure 2). The notable treatment efficiency was further validated by significant reductions of bacterial loads *in vivo*, and remarkable improvement of zebrafish survival rate on day 14 (Figure 9A). The results were comparable to the data of AgNO_3_ or other metal-based drugs combined with antimicrobials. For instance, the combinations of AgNO_3_ (1.5 mg/kg) and colistin (2.0 μg/kg), AgNO_3_ (6.0 mg/kg) and vancomycin (30.0 μg/kg), and auranofin (0.5–1.0 mg/kg) and colistin (2.0–8.0 mg /kg), led to significant reductions of bacterial loads in tissues (e.g., 0.87–3.5 log), and increased the survival rates of infected animals (e.g., mice, zebrafish) (17, 19, 21, 29). Colloidal bismuth subcitrate (10 mg/kg) not only serves as broad-spectrum metallo-β-lactamase inhibitors (39), but also can combine with azithromycin (4 mg/kg) or chloramphenicol (2 mg/kg) effectively against *P. aeruginosa* in mice (18). Iridium (III) alone exhibits robust bactericidal efficacy against *Staphylococcus aureus*, and shows low cytotoxicity and good biocompatibility even at a high dose of 256 mg/kg *in vivo* (40). These findings highlight metal-based approaches may be a powerful way to fight against antimicrobial resistance and multi-drug resistant bacteria in the future. However, the antibacterial efficiencies of colistin and AgNO₃, and other metal based strategies should be tested on more cultured fish (e.g., tilapia, catfish) to provide conclusive and scalable data for practical applicability in aquaculture.

The underlying mechanisms of silver (Ag) against pathogens remain unclear in the fish diseases research. For instance, Shaalan et al. (41) and Ghetas et al. (42) reported the antibacterial effects of silver against MDR fish pathogens using disc diffusion assays and transmission electron microscopy observations. Satomi et al. (43) investigated the inhibitory effects and therapeutic efficiency of AgNO_3_ (50.0–100.0 μg/L) against *Aureispira anguillae* isolated from Japanese eel leptocephali without biochemical or transcriptional data. Lack of sufficient data would hamper the practical application in the real-world aquaculture environments. Accordingly, the present study conducted an in-depth investigation into the bactericidal activity and potential mechanisms of the AgNO_3_ and colistin combination.

**Figure 10:**
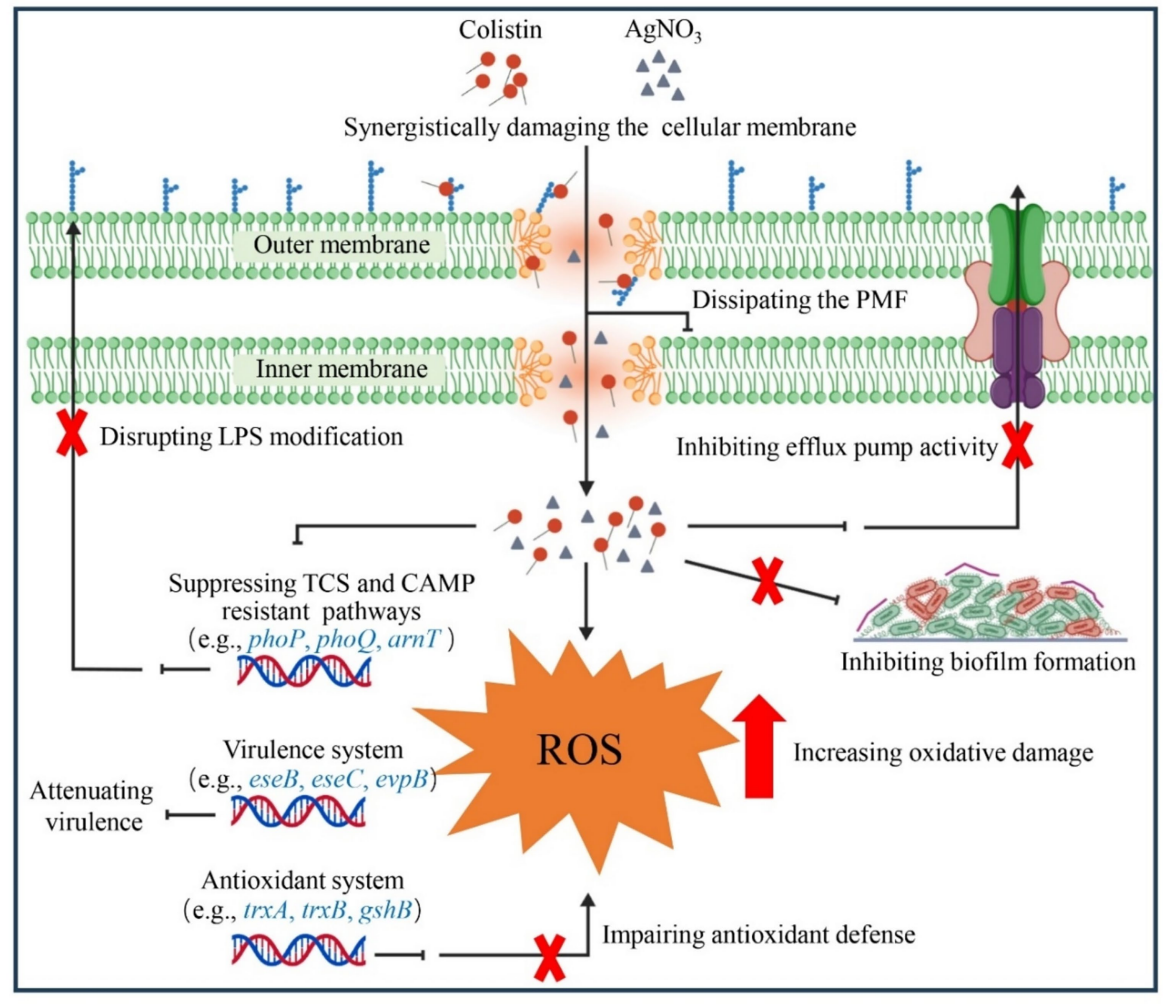
Schematic illustration of the synergistic interaction between AgNO_3_ and colistin.

A previous study reported that TrxR from *E. piscicida* shared 81% amino acid sequence similarity with that of *E. coli* and possessed similar conserved active residues (e.g., Cys136 and Cys139) (29). Silver (Ag) may also disrupt the Trx system of *E. piscicida*, resulting in oxidative stress. Accordingly, we observed that the combination of AgNO_3_ and colistin dramatically reduced the activity of TrxR (Figure 7C) and its mRNA expression level (Figure 6B), GSH content (Figure 7D), and expression of *gshA* and *gshB* (Figure 6B). Deletion of the *trx* genes (Figure 8) or the exogenous addition of 5.0 mM of the ROS scavenger NAC effectively abolished the synergetic interaction between AgNO_3_ and colistin in *E. piscicida*, leading to increased FICi values and bacterial survival (Figure 7G; Supplementary Figure S4). The data suggest that AgNO_3_ may synergistically enhance the bactericidal effect of colistin through directly inhibiting the Trx system and disrupting intracellular redox homeostasis (Figure 10). These findings are consistent with previous studies, showing the combinations of silver with other antibiotics (e.g., gentamicin) against MDR bacteria through inhibition of the Trx system and oxidative damage (19, 20, 44). Therefore, TrxR and the bacterial redox cascade may represent promising targets for antimicrobial therapies or resistance breakers against MDR bacteria.

Bacterial cationic antimicrobial peptides (CAMPs) resistance pathways, including those related to colistin, play a crucial role in the pathogenicity of various bacteria. For instance, both *arnB* and *ugd* mediate the CAMP resistance and contribute to *in vivo* colonization of *E. piscicida*, and loss of *arnB* or/and *ugd* impede the colistin resistance and virulence (45, 46). In *E. coli*, the development of colistin resistance enhances bacterial tolerance to host-derived antimicrobial peptides, and compromises the innate immune response and clearance (47, 48). Moreover, inhibition of the activities of MCR variants with silver, which directly contributes to colistin resistance, disrupts bacterial immune evasion and persistence abilities *in vivo* (21, 30, 49). The CAMPs pathway may represent an alternative target for silver containing drugs. In agreement with this speculation, AgNO_3_ combined with colistin dramatically suppressed the expression of genes involved in CAMPs pathways (Figure 6C), including those associated with two-component regulatory system (TCS) (e.g., *pmrA*, *prmB*, *phoP*, and *phoQ*), and LPS synthesis and modification (e.g., *ugd*, and *arnT*) (Figure 6C). It resulted in *E. piscicida* re-sensitized to colisitin, inhibition of type III and VI secretion systems, and enhanced bacterial clearance *in vivo* (Figure 9). Considering that *phoP* and *phoQ* are key members of the TCS, and positively regulate the expression of bacterial virulence genes (50, 51), their downregulation might also contribute to the inhibition of type III and VI secretion systems related genes (e.g., *eseB* and *evpP*) (Figure 6D), finally lowering *E. piscicida*’s virulence (Figure 9). Based on those findings, a schematic diagram was proposed (Figure 10), and the findings might offer valuable insights for future applications.

Metal homeostasis also plays a critical role in bacterial growth and survival, and has emerged as another potential target for the development of novel antimicrobials. For instance, colistin combined with natural flavonoids, or the zinc ionophore PBT2 restored antibiotic sensitivity in various MDR bacteria by disrupting the homeostasis of iron or zinc (30, 52). AgNO_3_ might also overcome the intrinsic colistin resistance in *E. piscicida* by dysregulating metal homeostasis. While inductively coupled plasma-mass spectrometry (ICP-MS) analysis revealed that the combination of AgNO_3_ and colistin did not significantly alter the intracellular contents of zinc, iron, copper, and calcium, but reduced intracellular silver content (Supplementary Figure S5). AgNO_3_ and colistin combination might not work through disruption of metal homeostasis, and further investigation is still needed to elucidate the precise bactericidal mechanisms to support future therapeutic application.

Moreover, the environmental and health risks of silver (including AgNO_3_) should not be ignored. Toxicological studies revealed that silver can accumulate in cultured fish species and induce chronic toxicity, including common carp (*Cyprinus carpio*) (53), *Oreochromis mossambicus* (54) and *Mytilus galloprovincialis* (55), posing a potential threaten to the safety of aquaculture and consumption. Moreover, high concentration of silver (≥500 μg/L) also reduced photosynthetic efficiency of the phytoplankton, and inhibited their growth (56, 57). Notably, most of the toxicological studies were carried out at higher concentrations and shorter exposure periods (58, 59), and optimal level of silver or other metal based compounds could cause no negative effects on tested fish (60, 61). For instance, AgNO_3_ (1.5–6.0 mg/kg) caused negligible toxicity to mice and mammalian cells (19–21), and no apparent clinical toxicity and metabolic changes happened in humans after daily ingestion of 100–480 μg of AgNPs for 14 days (62). AgNPs (4 to 64 μg/L) neither disrupted the phytoplankton community structure nor reduced any biomass (63), and even enhanced plankton-mediated carbon cycle at 10 and 100 μg/L (64). Specifically, Shaalan et al. (25) and Zhang et al. (65) found that AgNPs (100 μg/L), and AgNPs@C-dots (9.5 μg/mL) can safely treat infected rainbow trout (*Oncorhynchus mykiss*) and zebrafish with no residue silver in the muscles (below the detection limit, < 2 ng/g) at day 35 and 30, respectively. Dietary supplementation of 15 μg/kg AgNP even strengthened *Labeo rohita*’s innate immune and antioxidant systems (66).

The above contradictory results might be due to that Ag^+^ is released much lower from AgNPs, which contributes to lower bioavailability and toxicity in fish (53, 55). Thereby, AgNO_3_ would be transformed into AgNPs, and then mixed with antibiotics for bath treatments or dietary supplementation to treat diseased fish. The potential effects of silver is complex in aquatic organisms, and may depend on the treated concentration, typology, and species of tested organisms (67, 68), and future studies should focus on more aspects such as expanding the toxicological scope and duration, optimizing the administrated doses and delivery methods, developing novel additives simultaneously to enhance synergistic effects and minimize the potential risks, and establishing guidelines for the permissible limit of silver in aquaculture products for human consumption.

## 5 Conclusion

AgNO_3_ is an effective adjuvant that enhances the bactericidal effects of colistin in treating the intrinsically colistin resistant fish pathogen *E. piscicida*. The combination of AgNO_3_ and colistin acts synergistically to disrupt bacterial redox homeostasis and CAMP pathways. These actions exacerbate oxidative stress and cellular damage, while simultaneously reducing bacterial virulence *in vivo*. The collective findings support the repurposing of AgNO_3_ as a potent, non-antibiotic adjuvant for use alongside colistin to eradicate naturally colistin-resistant *E. piscicida*. This combination strategy may offer promising and practical approach for controlling MDR pathogens in aquaculture.

## Data Availability

The original contributions presented in the study are included in the article/Supplementary material, further inquiries can be directed to the corresponding authors.
