# Correction: Silver nitrate enhances antibacterial effect of colistin against intrinsic colistin resistant *Edwardsiella piscicida*

**DOI:** 10.3389/fvets.2026.1805831

**Published:** 2026-03-06

**Authors:** Frontiers Production Office

**Affiliations:** Frontiers Media SA, Lausanne, Switzerland

**Keywords:** fish diseases, antimicrobial resistance, silver ions, colistin, synergistic effect

Reviewer Ramsha Hafeez was erroneously assigned to affiliation “Lahore Pharmacy College, Pakistan.”

This affiliation has now been removed for Dr Hafeez, and has been corrected to:

“Lahore College for Women University, Lahore, Pakistan.”

The original version of this article has been updated.

