# Supplementary material for: Silver nitrate enhances antibacterial effect of colistin against intrinsic colistin resistant Edwardsiella piscicida
Source: Front Vet Sci. 2025 Oct 29;12:1679761. doi: 10.3389/fvets.2025.1679761 (PMC12604994; doi:10.3389/fvets.2025.1679761)
Supplement: Supplementary file 1 [file Data_Sheet_1.docx]

**Supplementary Materials**

**Text S1-2, table S1-3 and Figure S1-7**

**Silver nitrate enhances antibacterial effect of colistin against intrinsic colistin resistant *Edwardsiella piscicida***

Yangbin Shi^1^, Luyu Mei^1^, Zubair Ahmed Laghari^2^, Caiyi Tu^1^, Yajing Pan^1^, He Zhang^3,*^, Yongliang Lou^1,*^, Jinfang Lu^1,**^

^1^Wenzhou Key Laboratory of Sanitary Microbiology, Key Laboratory of Laboratory Medicine, Ministry of Education, China, School of Laboratory Medicine and Life Sciences, Wenzhou Medical University, Wenzhou, Zhejiang 325035, China

^2^ Department of Veterinary Parasitology, Sindh Agriculture University, Tandojam, 70060, Sindh, Pakistan

^3^Zhejiang Provincial Key Laboratory for Subtropical Water Environment and Marine Biological Resources Protection, National and Local Joint Engineering Research Center of Ecological Treatment Technology for Urban Water Pollution, College of Life and Environmental Sciences, Wenzhou University, Wenzhou, Zhejiang 325035, China

* Correspondence: Yongliang Lou, lyl@wmu.edu.cn or He Zhang, zhanghe@wzu.edu.cn

Jinfang Lu, jflu@wmu.edu.cn

**This supplementary information contains:**

⚫ 12 Pages

⚫ 2 Texts

⚫3 Tables

⚫ 7 Figures

**Text list:**

Text S1 Chemicals and reagents

Text S2 Inductively coupled plasma-mass spectrometry (ICP-MS) analysis

**Table list:**

**Table S1** Bacterial strains used in this work.

**Table S2** A list of primers used for qRT-PCR.

**Table S3** The administered doses of adjuvant and colistin against bacterial infection

**Figure list:**

**Fig. S1** The relative expression levels of swimming motility related genes at 24 hours after

treatment by AgNO_3_ or colistin alone, and the combination of AgNO_3_ and colistin.

**Fig. S2** AgNO_3_ potentiates colistin’s membrane damage activity and dissipates the proton

motive force in *E. piscicida* isolates ZX-1 and LY-2019.

**Fig. S3** AgNO_3_ enhances efflux pump inhibition, facilitates intracellular colistin accumulation

in *E. piscicida* isolates.

**Fig. S4** Isobolograms of *E. piscicida* isolates ZX-1 and LY-2019 treated by colistin + AgNO_3_ + NAC.

**Fig. S5** Intracellular contents of metals in *E.piscicida* PPD130/91.

**Fig. S6** Combined AgNO_3_ and colistin treatment induced ROS in *E. piscicida* isolates ZX-1

and LY-2019.

**Fig. S7** Bacterial loads in the liver and spleen of infected zebrafish at 48 hours after treated

by colistin (8.0 mg/kg), AgNO_3_ (1.5 mg/kg), or their combination (8.0 + 1.5 mg/kg).

**Text S1. Chemicals and reagents**

Colistin sulfate and the 1-N-phenylnaphthylamine (NPN) probe were obtained from MedChem Express (Shanghai, China). Silver nitrate (AgNO_3_) and the Thioredoxin Reductase assay kit were purchased from Sigma Aldrich (St. Louis, MO, USA), and Solarbio (Beijing, China), respectively. Propidium iodide (PI), Hoechst 33342, N-acetyl-L-cysteine (NAC), and the 2′, 7′-dichlorodihydrofluorescein diacetate (DCFH-DA) probe, along with commercial kits for the detection of malondialdehyde (MDA) content and the activities of superoxide dismutase (SOD), and catalase (CAT), were purchased from Beyotime (Shanghai, China). The 3,3'-dipropylthiacarbocyanine iodide [DiSC_3_(5)] probe and the LIVE/DEAD BacLight Bacterial Viability Kit were obtained from Thermo Fisher Scientific (Shanghai, China). The PrimeScript™ RT reagent kit with gDNA Eraser, Bacteria RNA Extraction Kit, and SYBR Mix were purchased from Takara (Liaoning, China), Vazyme (Nanjing, China) and Qingke (Beijing, China), respectively. Tryptic Soy Broth (TSB) media and Cation-adjusted Mueller-Hinton Broth (CAMHB) were purchased from BD Biosciences (San Jose, CA, USA) and Hopebio (Qingdao, China), respectively.

**Text S2. Inductively coupled plasma-mass spectrometry (ICP-MS) analysis**

ICP-MS (Agilent 7500a; Agilent Technologies) was performed to determine the intracellular concentrations of the elements silver (Ag), iron (Fe), zinc (Zn), magnesium (Mg), and calcium (Ca) as described in previous studies (1, 2). Briefly, *E. piscicida* cultures in the exponential-phase were diluted to an OD_540_ ≈ 0.5, and then treated with colistin, AgNO_3_ alone, or their combination for 1 hour. Bacterial pellets were collected by centrifugation (8,000 rpm, 5 minutes), and lysed overnight in a mixture of 6 mL of HNO_3_ and 2.0 mL of H_2_SO_4_, using indium (1.0 μg/mL) as an internal standard. The supernatant was then collected by centrifugation (14,000 rpm, 10 minutes), and filtered through 0.2 µm filters for ICP-MS analysis.

References

1. Xia Y, Wei X, Gao P, Wang C, de Jong A, Chen JHK, et al. Bismuth-based drugs sensitize *Pseudomonas aeruginosa* to multiple antibiotics by disrupting iron homeostasis. *Nat Microbiol*. (2024) 9:2600-2613. doi: 10.1038/s41564-024-01807-6.

2. Yu Y, Zhao H, Lin J, Li Z, Tian G, Yang Y, et al. Repurposing non-antibiotic drugs auranofin and pentamidine in combination to combat multidrug-resistant Gram-negative bacteria. *Int J Antimicrob Agents*. (2022) 59:106582. doi:10.1016/j.ijantimicag.2022.106582.

| **Strain or plasmid** | **Genotype and description** | **Source** | |
| --- | --- | --- | --- |
| ***Edwardsiella piscicida*** |  | |  |
| PPD130/91 | Wild-type, Col^r^ | | Lu et al., 2015 |
| △*trxA* | PPD130/91 with in-frame deletion mutation of *trxA*, Col^r^ | | Lu et al., 2025 |
| △*trxB* | PPD130/91 with in-frame deletion mutation of *trxB*, Col^r^ | |  |
| △*trxC* | PPD130/91 with in-frame deletion mutation of *trxC*, Col^r^ | |  |
| △*trxA*△*trxC* | PPD130/91 with in-frame deletion mutation of *trxA* and *trxC*, Col^r^ | |  |
| △*trxA*△*trxB*△*trxC* | PPD130/91 with in-frame deletion mutation of *trxA*, *trxB* and *trxC*, Col^r^ | |  |
| ZX-1 | Wild-type，Col^r^ | |  |
| LY-2019 | Wild-type，Col^r^ | |  |

Table S1 Bacterial strains used in this work.

Col, colistin; Superscripts: r, resistance.

References

1. Lu J, Wang W, Wang G, Zhang H, Zhou Y, Gao Z, et al. *Edwardsiella tarda* EscE (Orf13 protein) is a type III secretion system-secreted protein that is required for the injection of effectors, secretion of translocators, and pathogenesis in fish. *Infect Immun*. (2015) 84:2-10. doi:10.1128/IAI.00986-15.

2. Lu J, Shi Y, Pan Y, Grossart, HP, Mei L, Xie D, et al. 2025. Repurposing auranofin combined with colistin to effectively combat fish-pathogenic *Edwardsiella piscicida*. *Aquac Rep*. (2025) 42:1-14. doi:10.1016/j.aqrep.2025.102830.

Table S2 A list of primers used for qRT-PCR.

| primer | sequence（5’→3’） |
| --- | --- |
| EP-16s-qPCR-For | ACTGAGACACGGTCCAGACTCCTAC |
| EP-16s-qPCR-Rev | TTAACGTTCACACCTTCCTCCCTAC |
| trxA-qPCR-For | GTGCGGTCCGTGCAAGATGATT |
| trxA-qPCR-Rev | AACAGCAGCAGCGTCGGAATAC |
| trxB-qPCR-For | ATCGACCGCCTGATGGACAAAG |
| trxB-qPCR-Rev | GGACTGTACCTGAATGTAGCCGTTA |
| trxC-qPCR-For | TCATCCAGCCAGGCTTCAAAC |
| trxC-qPCR-Rev | TCCGCATTCGCAGCATTCC |
| gshA-qPCR-For | TTGCTCTGCGACTTATTGGTGTA |
| gshA-qPCR-Rev | GCCATCTGTGGTTCCTTCCTG |
| gshB-qPCR-For | CTGCTGGATGCGGAAGATGGA |
| gshB-qPCR-Rev | GCACCCTGATCGTCAACAAACC |
| acrA-qPCR-For | CGGACGGACTTCTGCGATAC |
| acrA-qPCR-Rev | TGGCGGCGATACTGCTGTT |
| acrB-qPCR-For | GCTGTCGGTATAGTGGTGAGT |
| acrB-qPCR-Rev | ATCTATCGCCAGTTCTCCATCA |
| tolC-qPCR-For | AGGTTCTGCTTGGCGTTGT |
| tolC-qPCR-Rev | GAATGTCCGCTCCTCCTTCA |
| emrB-qPCR-For | TGCGACGACTGGTCACCTT |
| emrB-qPCR-Rev | GCGGTATAGCCATACACCTCCT |
| mdtl-qPCR-For | ATTGAGCTGTCCGTCGCCTA |
| mdtl-qPCR-Rev | ACCGCCATACCGACCAACA |
| phoP-qPCR-For | AGAAGACGGCATGAGCATGA |
| phoP-qPCR-Rev | TGGAAGGGTTTGGTGACATAGT |
| phoQ-qPCR-For | GCGATGGTGTTTCTGCTCTC |
| phoQ-qPCR-Rev | GGCGATAGGTGGTCTTGTCA |
| basR-qPCR-For | GGACTGTGCAACAACGCTCTG |
| basR-qPCR-Rev | GGAGTAGAGATCCTGCTGGAGAAC |
| basS-qPCR-For | CGATCACCCGTACCCTCAACCA |
| basS-qPCR-Rev | CGCTCTGCTCCATCAGCTCAAG |
| arnT-qPCR-For | GTATTACCTGCCGATCCTCCTTC |
| arnT-qPCR-Rev | CTGAACAGCAGAAATGGCATCAC |
| ugd-qPCR-For | GCTCAACAGCCGTCAGATTATTG |
| ugd-qPCR-Rev | AAGTTATCCGAACCGCTCTTCA |
| eseB-qPCR-For | CCAACGGCTACGACAACAAC |
| eseB-qPCR-Rev | ATTAGCCACCTGCTGGGAGT |
| eseC-qPCR-For | ATAGACTGGTACTTTTCCGC |
| eseC-qPCR-Rev | TTGGCAAAATCTTAATCGGC |
| eseD-qPCR-For | AAAGTCTTTATCGATGGCGT |
| eseD-qPCR-Rev | GATGGTTCAGCTTGGAGAG |
| evpB-qPCR-For | ATGACAGTGACTCCGCCAAT |
| evpB-qPCR-Rev | CATCCGAGCCGACCACATA |
| evpC-qPCR-For | TACAAATTTACGCATGTCGC |
| evpC-qPCR-Rev | GATCCTGGGGAACGTATTC |
| evpP-qPCR-For | AGTCCTAAAGATTCCCGTCT |
| evpP-qPCR-Rev | AATATAGAACTGTGTGGCCC |
| ompR-qPCR-For | AAACTCGCCGCTGGTCAA |
| ompR-qPCR-Rev | AGAGGACGCCGTGATTAACTT |

Table S3 The administered doses of adjuvant and colistin against bacterial infection

|  | Microorganism | Adjuvant (dose) | Colistin (dose) | Tested animal | Reference |
| --- | --- | --- | --- | --- | --- |
| 1 | *Salmonella SL1344* | Dephostatin (2.5 mg/kg) | 10 mg/kg | mouse | (1) |
| 2 | *Salmonella S281* | tetrandrine (15 mg/kg) | 10 mg/kg | mouse | (2) |
| 3 | *S. typhimurium* | 7,8-DHF (5 mg/kg) | 5 mg/kg | mouse | (3) |
| 4 | *E. coli* | flavomycin (16 mg/kg) | 2 mg/kg | mouse | (4) |
| 5 | *Salmonella SB05* | berberine (80mg/kg) + EDTA (10 mg/kg) | 8mg/kg | mouse | (5) |
| 6 | *K. pneumonia* | AgNO3 (1.5 mg/kg) | 2 mg/kg | mouse | (6) |
| 7 | *E. piscicida* | auranofin (2.0 mg/kg) | 8 mg/kg | zebrafish | (7) |
| 8 | *E. piscicida* | AgNO3 (1.5 mg/kg) | 8 mg/kg | zebrafish | This study |

References

1. Tsai CN, MacNair CR, Cao MPT, Perry JN, Magolan J, Brown ED, et al. Targeting two-component systems uncovers a small-molecule inhibitor of *Salmonella* virulence. *Cell Chem Biol*. (2020) 27:793-805.e7. doi: 10.1016/j.chembiol.2020.04.005.

2. Yi K, Liu S, Liu P, Luo X, Zhao J, Yan F, et al. Synergistic antibacterial activity of tetrandrine combined with colistin against MCR-mediated colistin-resistant *Salmonella*. *Biomed Pharmacother*. (2022) 149:112873. doi: 10.1016/j.biopha.2022.112873.

3. Zhong Z, Zhou S, Liang Y, Wei Y, Li Y, Long T, et al. Natural flavonoids disrupt bacterial iron homeostasis to potentiate colistin efficacy. *Sci Adv*. (2023) 9:4205. doi:10.1126/sciadv.adg4205.

4. Huang Y, Zhu Y, Yue H, Liu Y, Deng L, Lv L, et al. Flavomycin restores colistin susceptibility in multidrug-resistant Gram-negative bacteria. *mSystems*. (2024) 9:e0010924. doi: 10.1128/msystems.00109-24.

5. Cui X, Liu X, Ma X, Li S, Zhang J, Han R, et al. Restoring colistin sensitivity in colistin-resistant *Salmonella* and *Escherichia coli*: Combinatorial use of berberine and EDTA with colistin. *mSphere*. (2024) 9:e0018224. doi: 10.1128/msphere.00182-24.

6. Zhang Q, Wang R, Wang M, Liu C, Koohi-Moghadam M, Wang H, et al. Re-sensitization of *mcr* carrying multidrug resistant bacteria to colistin by silver. *Proc Natl Acad Sci U S A*. (2022) 119: e2119417119. [doi:10.1073/pnas.2119417119](%20https:/doi.org/10.1073/pnas.2119417119).

7. Lu J, Shi Y, Pan Y, Hans-Peter Grossart, Mei L, Xie D, et al. Repurposing auranofin combined with colistin to effectively combat fish-pathogenic *Edwardsiella piscicida*. *Aquacult Rep*. (2025) 42:102830. doi:10.1016/j.aqrep.2025.102830.

**
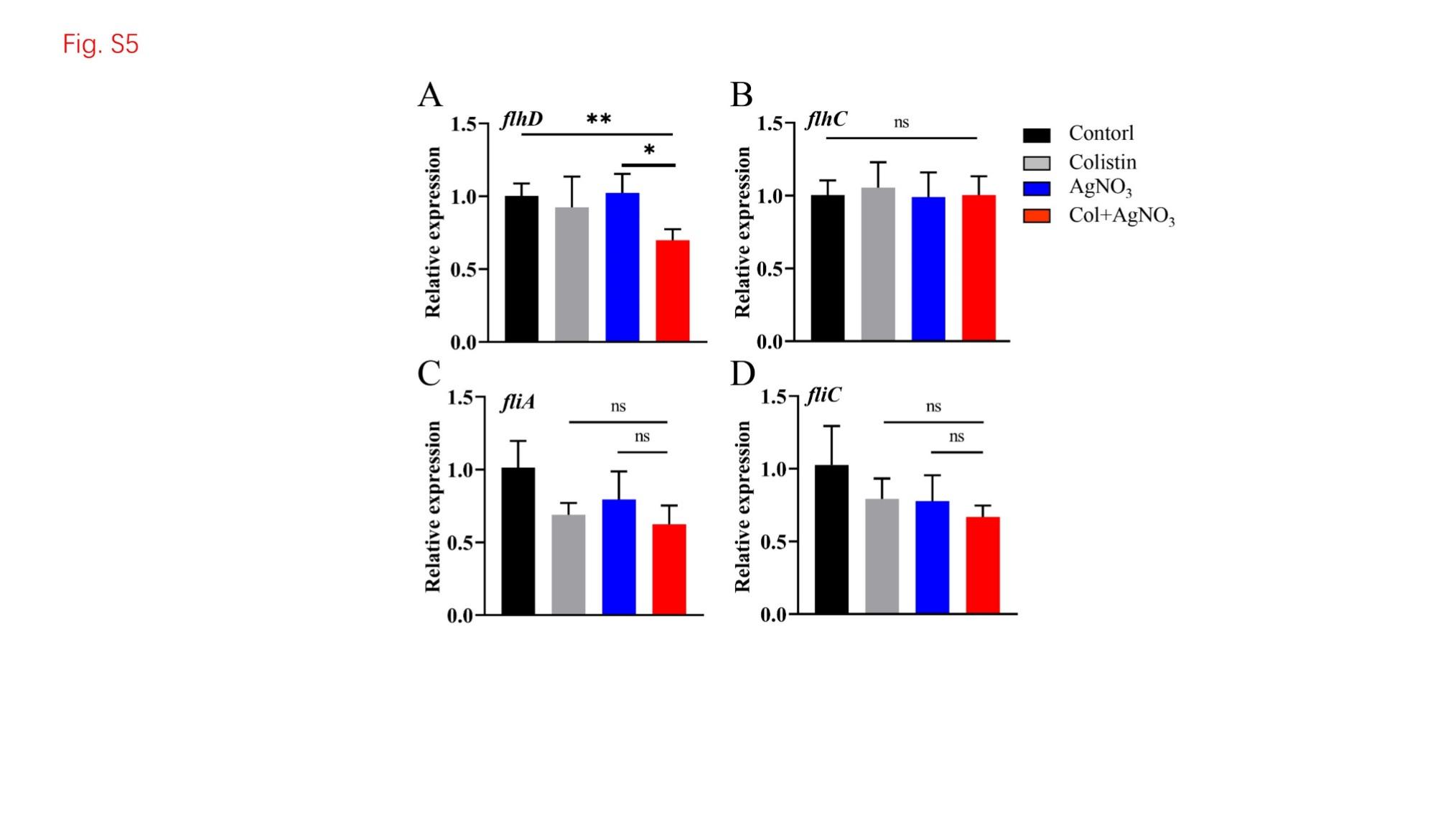
**

**Figure S1. The relative expression levels of swimming motility related genes at 24 hours after treatment by AgNO_3_ or colistin alone, and the combination of AgNO_3_ and colistin.** (A) *flhD*. (B) *flhC*. (C) *fliA*. (D) *fliC*. The values are expressed as mean ± SD (n=4), and statistical differences were tested by one-way ANOVA analysis. (* *p* < 0.05, ** *p* < 0.01).

**
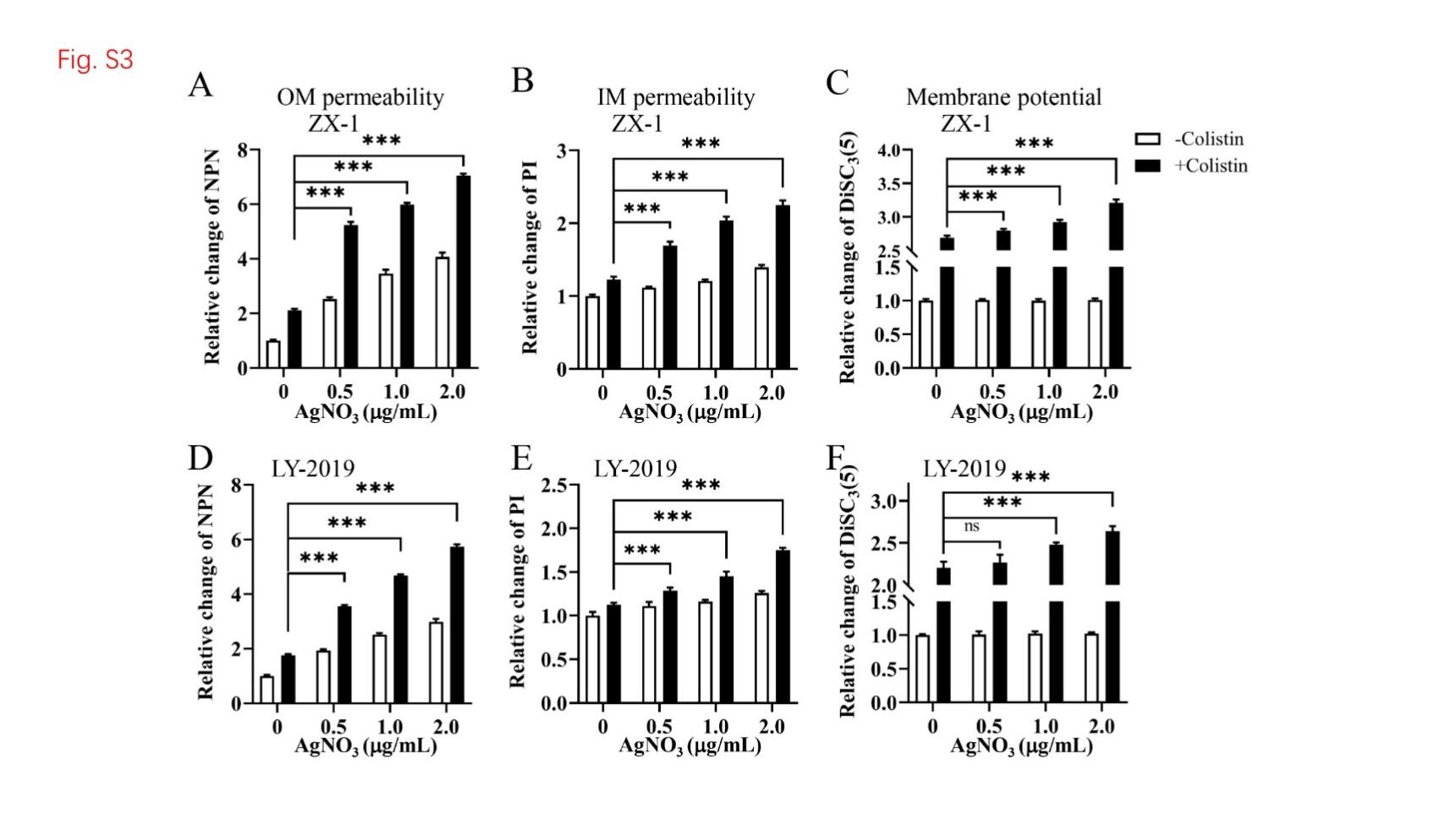
**

**Figure S2 AgNO_3_ potentiates colistin’s membrane damage activity and dissipates the proton motive force in *E. piscicida* isolates ZX-1 and LY-2019. (A)** and **(D)** AgNO_3_ potentiates the OM permeability in ZX-1 and LY-2019. **(B)** and **(E)** AgNO_3_ potentiates the IM permeability in ZX-1 and LY-2019. **(C)** and **(F)** AgNO_3_ dissipates the proton motive force in ZX-1 and LY-2019. The values are expressed as mean ± SD (n=4), and statistical differences were tested by two-way ANOVA analysis. (*** *p* < 0.001).

**
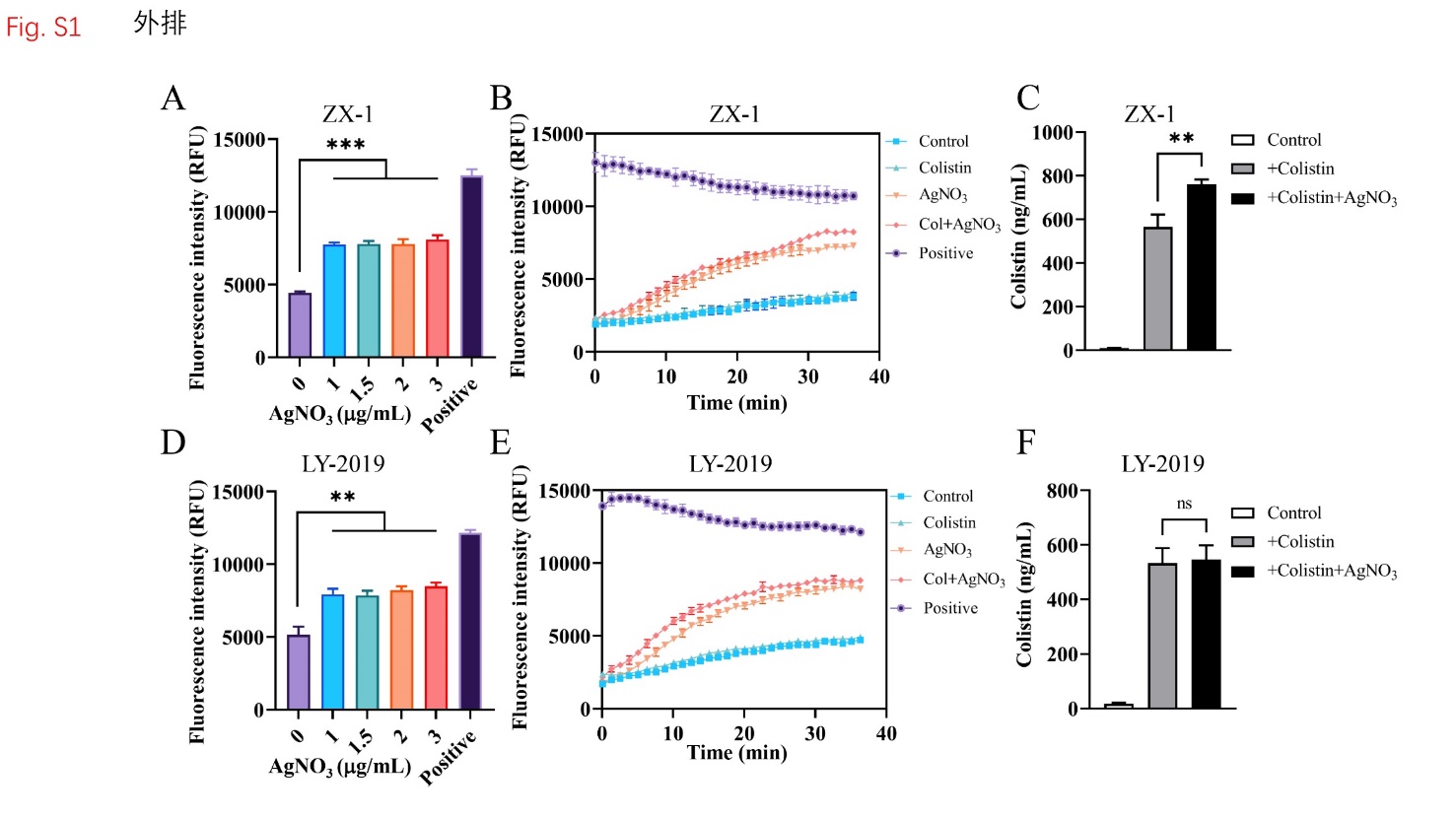
**

**Figure S3 AgNO_3_ enhances efflux pump inhibition, facilitates intracellular colistin accumulation in *E. piscicida* isolates.** **(A-B)** AgNO_3_ inhibits the efflux activity in ZX-1. **(C)** Intracellular content of colistin in isolate ZX-1. **(D-E)** AgNO_3_ inhibits the efflux activity in LY-2019. **(F)** Intracellular content of colistin in isolate LY-2019. The efflux activity was assessed by detection of the accumulation of Hoechst 33342. The heat-inactivated cells were used as positive control. The values are expressed as mean ± SD (n=4), and statistical differences were tested by one-way ANOVA analysis. (** *p* < 0.01, *** *p* < 0.001).


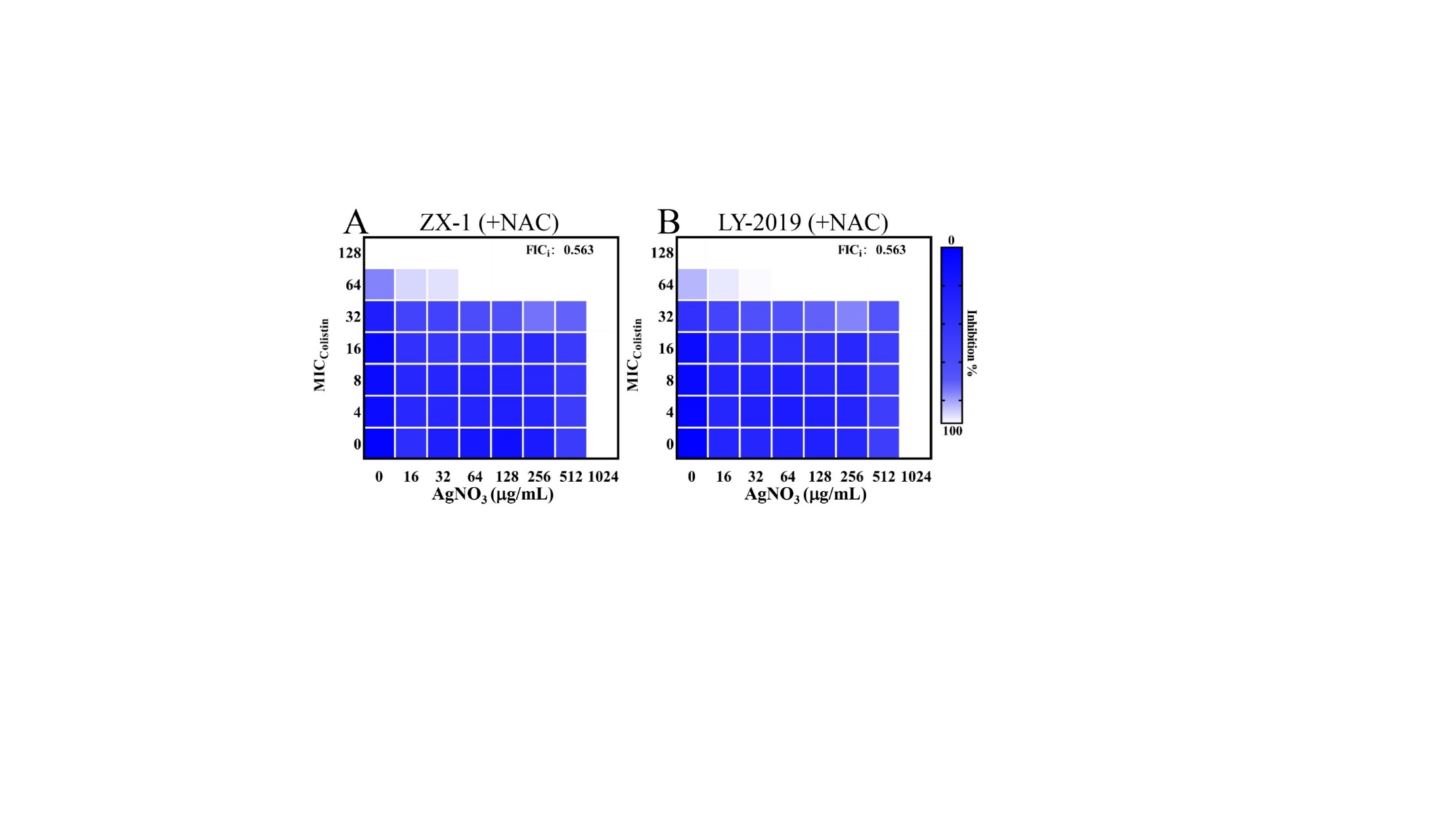


**Figure S4** Isobolograms of *E. piscicida* isolates ZX-1 (**A**) and LY-2019 (**B**) treated by colistin + AgNO_3_ + NAC.

**
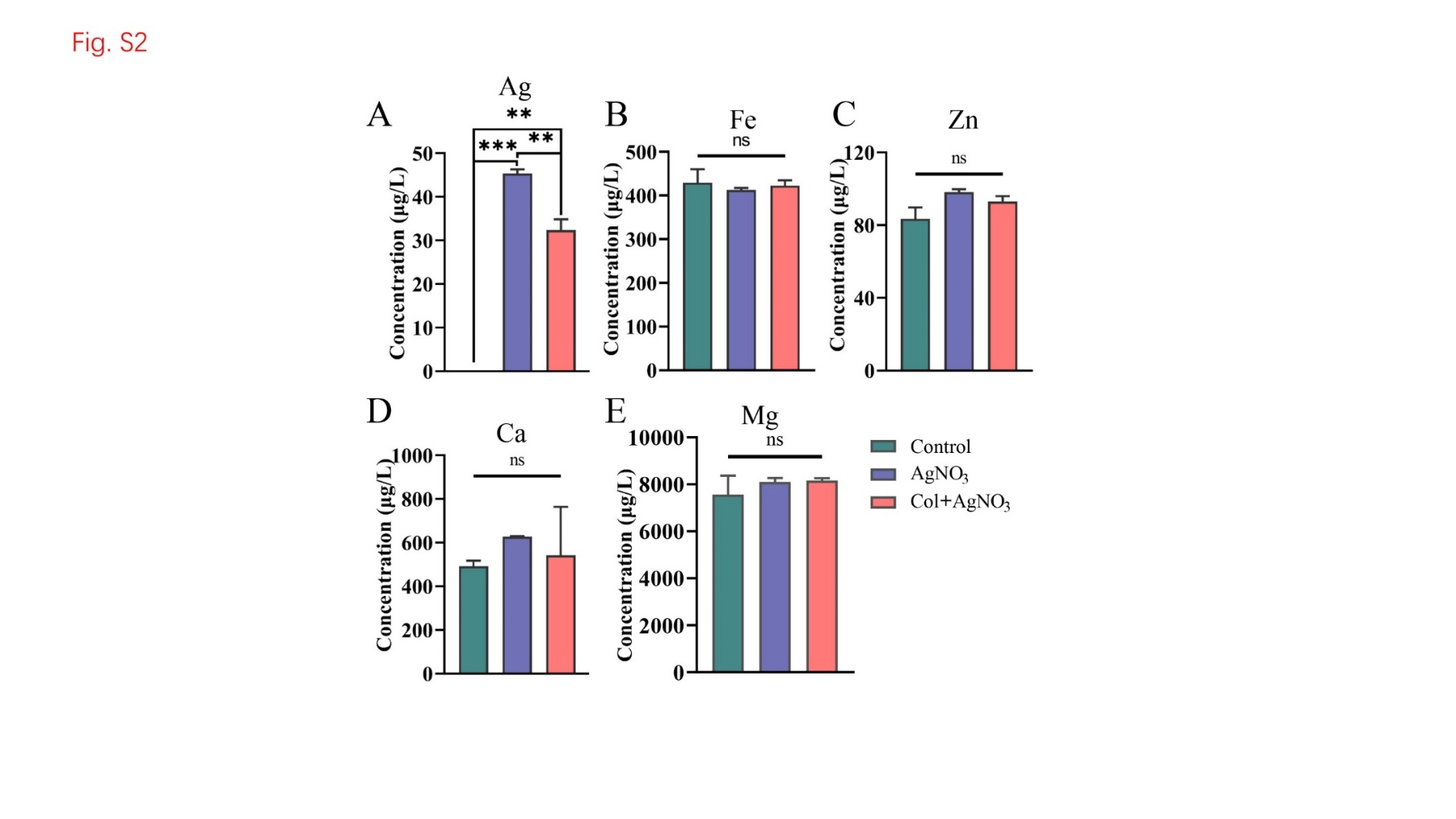
**

**Figure S5** **Intracellular contents of metals in *E.piscicida* PPD130/91.** Intracellular contents of silver, magnesium, iron, zinc, and calcium in *E. piscicida* PPD130/91. The values are expressed as mean ± SD (n=3), and statistical differences were tested by one-way ANOVA analysis. (** *p* < 0.01, *** *p* < 0.001).

**
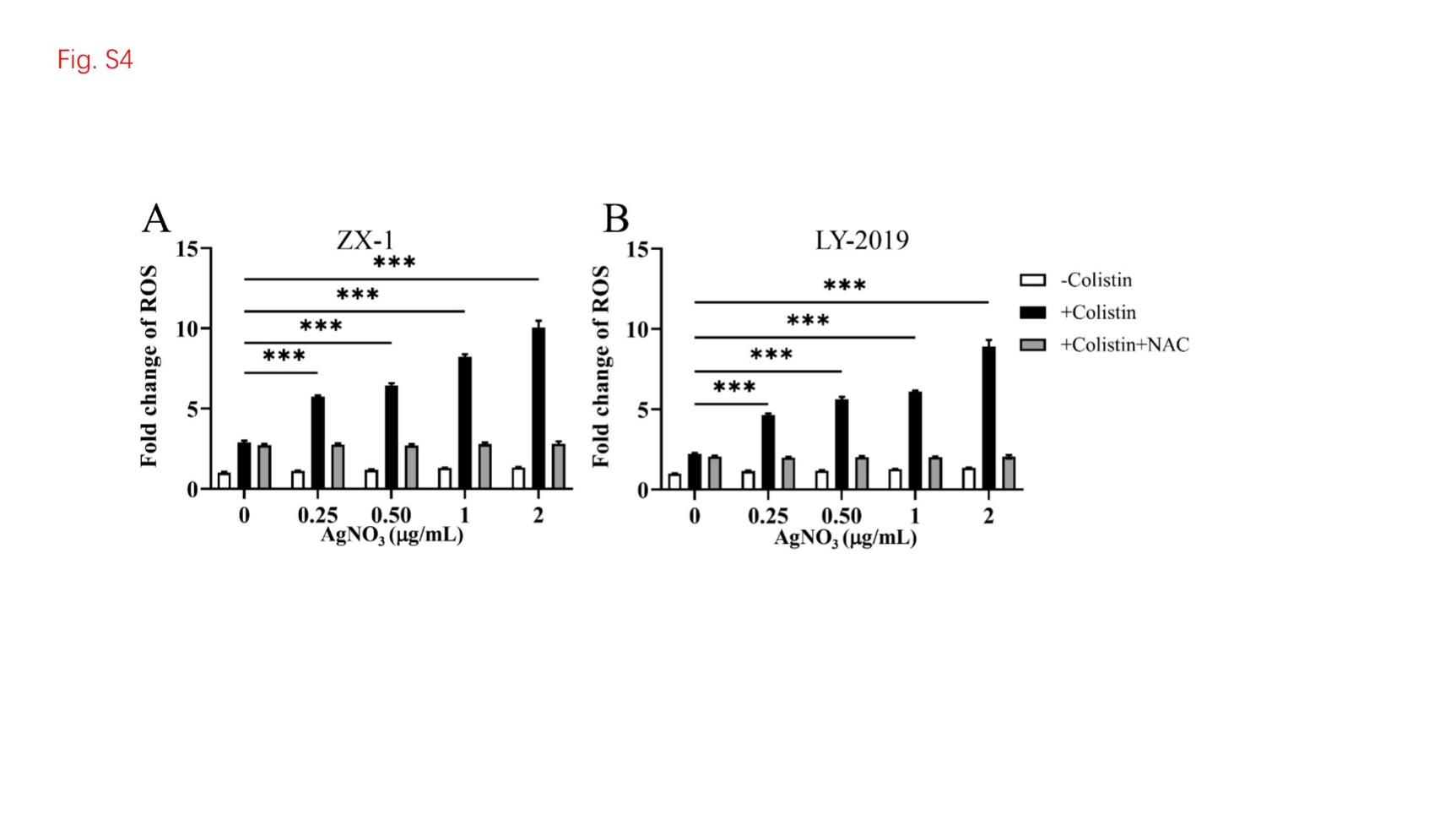
**

**Figure S6. Combined AgNO_3_ and colistin treatment induced ROS in *E. piscicida* isolates ZX-1 and LY-2019.** Relative ROS levels in ZX-1 and LY-2019 after exposure to increasing concentrations of AgNO_3_, colistin, AgNO_3_ + colistin (16.0 µg/mL), and AgNO_3_ + colistin + NAC. **(A)** ZX-1. **(B)** LY-2019. The values are expressed as mean ± SD (n=4), and statistical differences were tested by two-way ANOVA analysis. (****p* < 0.001).

**
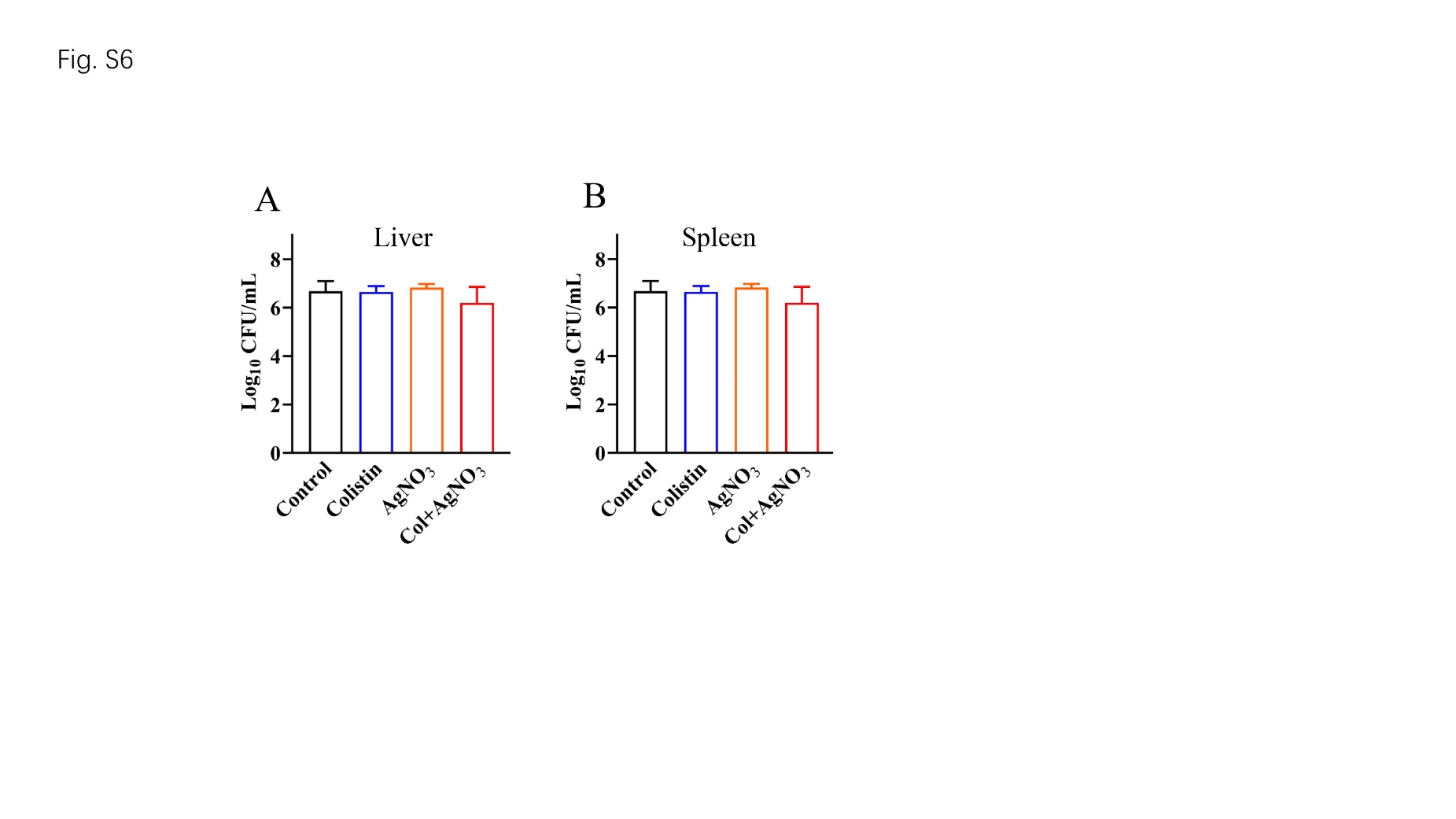
**

**Figure S7. Bacterial loads in the liver and spleen of infected zebrafish at 48 hours after treated by colistin (8.0 mg/kg), AgNO_3_ (1.5 mg/kg), or their combination (8.0 + 1.5 mg/kg)**. **(A)** liver. **(B)** spleen. The values are expressed as mean ± SD (n = 5).
